# Identification of novel conserved peptide uORF homology groups in Arabidopsis and rice reveals ancient eukaryotic origin of select groups and preferential association with transcription factor-encoding genes

**DOI:** 10.1186/1741-7007-5-32

**Published:** 2007-07-30

**Authors:** Celine A Hayden, Richard A Jorgensen

**Affiliations:** 1 Department of Plant Sciences, University of Arizona, Tucson, AZ 85721-0036, USA

## Abstract

**Background:**

Upstream open reading frames (uORFs) can mediate translational control over the largest, or major ORF (mORF) in response to starvation, polyamine concentrations, and sucrose concentrations. One plant uORF with conserved peptide sequences has been shown to exert this control in an amino acid sequence-dependent manner but generally it is not clear what kinds of genes are regulated, or how extensively this mechanism is invoked in a given genome.

**Results:**

By comparing full-length cDNA sequences from Arabidopsis and rice we identified 26 distinct homology groups of conserved peptide uORFs, only three of which have been reported previously. Pairwise *K*_*a*_/*K*_*s *_analysis showed that purifying selection had acted on nearly all conserved peptide uORFs and their associated mORFs. Functions of predicted mORF proteins could be inferred for 16 homology groups and many of these proteins appear to have a regulatory function, including 6 transcription factors, 5 signal transduction factors, 3 developmental signal molecules, a homolog of translation initiation factor eIF5, and a RING finger protein. Transcription factors are clearly overrepresented in this data set when compared to the frequency calculated for the entire genome (p = 1.2 × 10^-7^). Duplicate gene pairs arising from a whole genome duplication (ohnologs) with a conserved uORF are much more likely to have been retained in Arabidopsis (*Arabidopsis thaliana*) than are ohnologs of other genes (39% vs 14% of ancestral genes, p = 5 × 10^-3^). Two uORF groups were found in animals, indicating an ancient origin of these putative regulatory elements.

**Conclusion:**

Conservation of uORF amino acid sequence, association with homologous mORFs over long evolutionary time periods, preferential retention after whole genome duplications, and preferential association with mORFs coding for transcription factors suggest that the conserved peptide uORFs identified in this study are strong candidates for translational controllers of regulatory genes.

## Background

Upstream open reading frames (uORFs) are small open reading frames found in the 5' UTR of a mature mRNA, and can mediate translational regulation of the largest, or major, ORF (mORF). Regulation by uORFs has been studied in several individual transcripts demonstrating the importance of uORFs in such processes as polyamine production [1], amino acid production [2,3], and sucrose response [4], but the biological effect of uORFs in the vaste majority of transcripts of the genome is still unclear. Upstream start codons (uAUGs) occur in 20–30% of yeast, mammalian, and plant transcript 5' UTRs [5-7] therefore potentially thousands of genes are regulated in this manner.

The majority of characterized uORFs appear to act in an amino acid sequence-independent manner, regulating mORF translation by the uORF start codon nucleotide context, by the uORF length, or by the distance between the uORF stop codon and the mORF start codon, rather than by uORF-encoded peptides [8-11]. Some uORFs, however, do rely on peptide sequences to mediate translational regulation of the associated mORF, but few examples have been identified and characterized to date. In fungi and animals, a few genes have been shown to contain uORFs whose amino acid sequences are similar between two or more species [12-17], but only two cases, *CPA1 *[3] and *SAMDC1*/*AdoMetDC1 *[18], have demonstrated uORF sequence-dependent regulation. In plants two groups of genes, S-Adenosylmethionine decarboxylases (AdoMetDCs; EC 4.1.1.50) and group S basic region leucine zipper (bZIP) transcription factors, have been shown to contain uORFs with similar amino acids between monocots and dicots [19,20]. In the former group, mORF translational regulation is dependent on the sequence of the uORF peptide [1,4] and overexpression of the mORF in either group results in stunted or lethal phenotypes, suggesting that these genes play a critical role in growth and/or development. Indeed, AdoMetDC is required for polyamine synthesis, molecules that are implicated in essential plant functions such as cell division, embryogenesis, leaf, root, and flower development, and stress responses [21,22].

In general, it has been difficult to carry out genome-wide surveys of conserved peptide uORFs due to poor annotation of 5' UTRs. The availability of expressed sequence tags (ESTs) has improved exon and intron annotation of the genomic sequence, but they are relatively short and often do not predict the entire mRNA molecule, even when several ESTs overlap the same genomic region and can be assembled to predict one transcript. As there are very few introns in yeast transcripts, prediction of uORF conservation has been attempted in *S. cerevisiae *by analyzing genomic sequence upstream of predicted mORF start sites [23], but it is still not clear whether these uORFs are truly conserved (i.e., are under negative selection pressures), or are simply undergoing evolutionary drift. With the sequencing of the *Aspergillus nidulans *genome, comparison to *A. fumigatus *and *A. oryzae *has identified 38 uORFs with putatively conserved start and stop codon positions relative to the mORF, 14 of which are conserved in one of *Neurospora crassa, Fusarium graminaerum*, or *Magnaporthe grisea *[5], but the authors did not comment on whether the uORF amino acid sequences are also conserved.

With the emergence of large plant full-length cDNA sequence collections [24-26], it is now possible to adopt a comparative genomics approach to determine the prevalence of conserved amino acid uORFs in the genome and the persistence of these elements throughout eukaryotic evolution. Because rice and Arabidopsis shared a common ancestor 140–200 million years ago (Mya) [27-29], sequence similarity retained over this amount of time provides good candidates for truly conserved peptide uORF sequences. In this study we have used *Oryza sativa *(rice) and *Arabidopsis thaliana *(Arabidopsis) full-length cDNA sequence collections to estimate the incidence of conserved peptide uORFs in the rice and Arabidopsis genomes, to determine the prevalence of uORFs within regulatory genes, and to compare evolutionary rates for uORFs versus mORFs. By examining more distantly related sequences, we posit an ancient origin for select uORFs and we provide evidence for one mechanism by which uORFs can arise within genes.

## Results

### Identification of conserved peptide uORFs by comparison of rice and Arabidopsis transcripts

**Figure 1:**
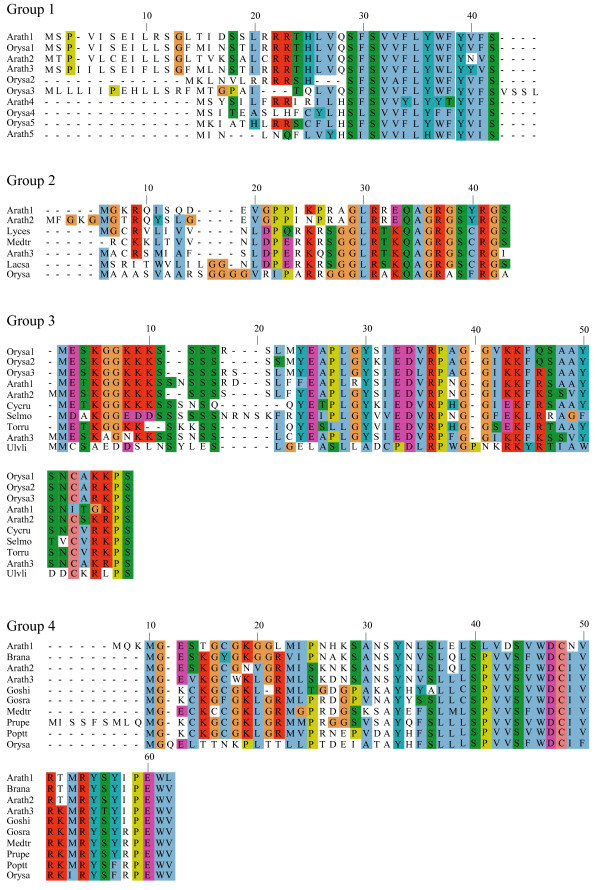
**Alignments of plant uORF homology groups 1–4**. Plant sequences were aligned using ClustalW v. 1.82 and displayed using Jalview. See main text for abbreviated species names and Genbank accession number, cDNA clone number, or genome identifier.

**Figure 2:**
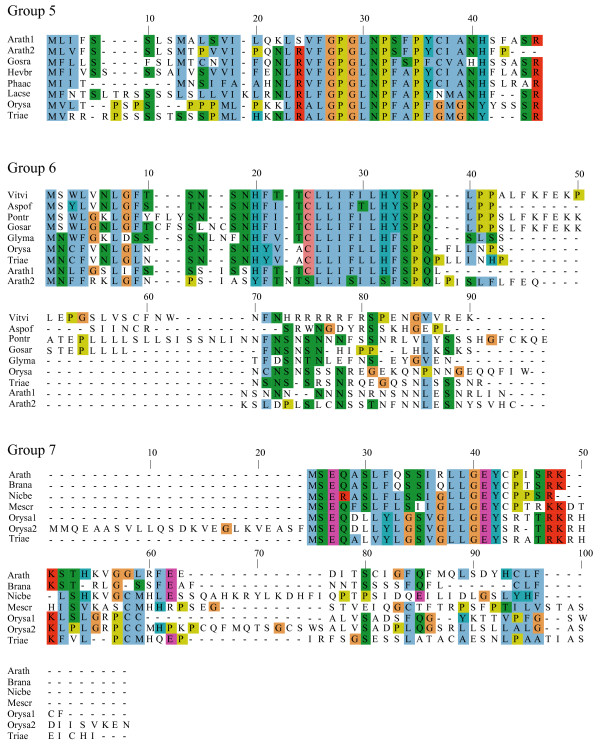
**Alignments of plant uORF homology groups 5–7**. Details as in Figure 1.

**Figure 3:**
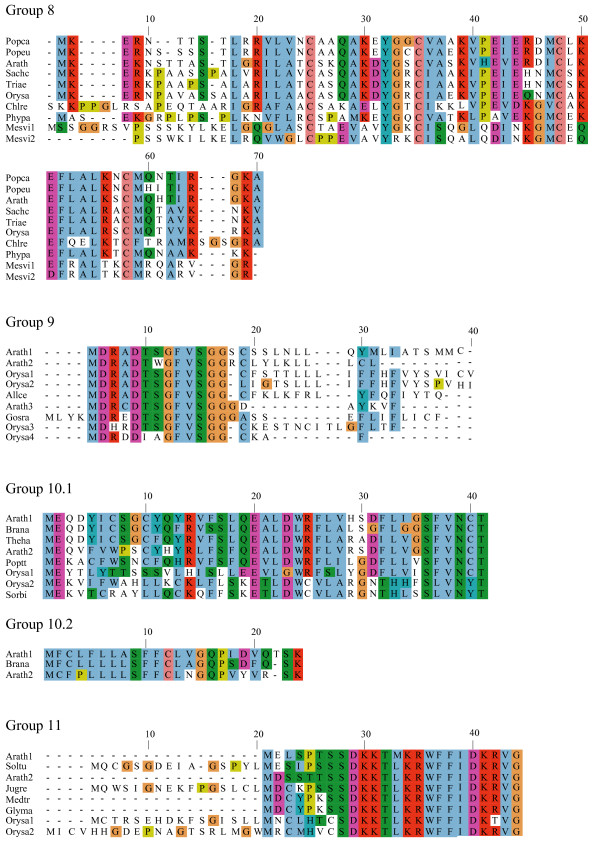
**Alignments of plant uORF homology groups 8–11**. Details as in Figure 1. Decimal places in the group number indicate multiple conserved uORFs in a given 5' UTR.

**Figure 4:**
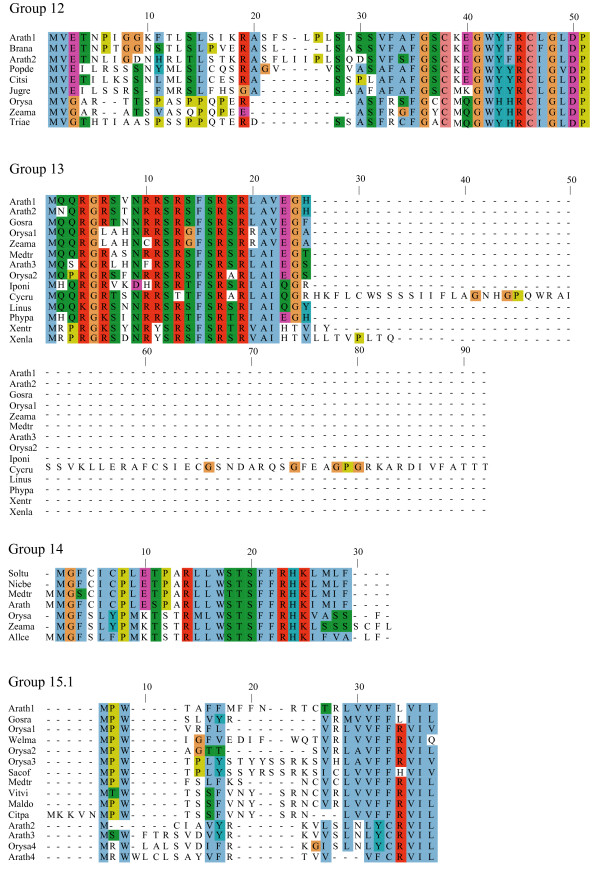
**Alignments of plant uORF homology groups 12–15.1**. Details as in Figure 1. Decimal places in the group number indicate multiple conserved uORFs in a given 5' UTR.

**Figure 5:**
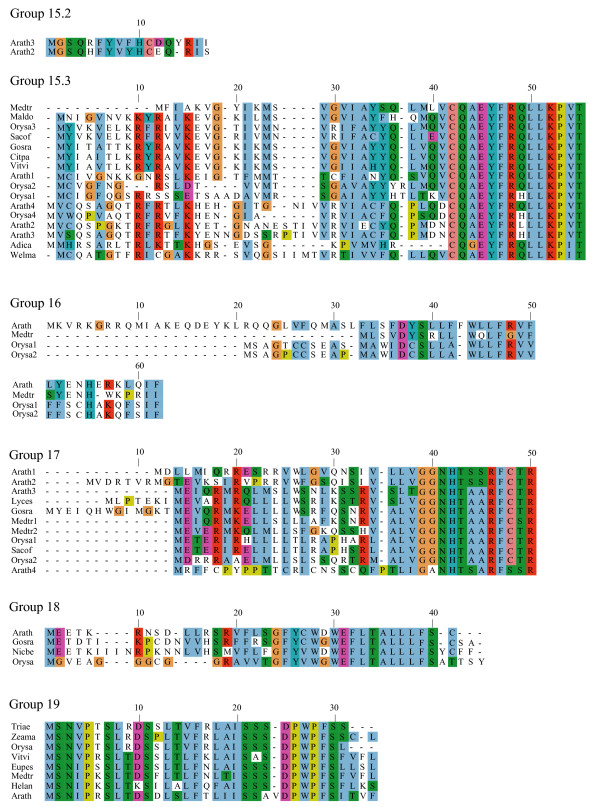
**Alignments of plant uORF homology groups 15.2–19**. Details as in Figure 1. Decimal places in the group number indicate multiple conserved uORFs in a given 5' UTR.

To identify conserved peptide uORFs, we developed "uORF-Finder", a Perl program that first compares the mORF amino acid sequence of each cDNA from one collection with the mORF sequences of another species' collection to identify putative mORF homologs, and then compares the uORFs in the 5' UTRs of the two paired sequences to identify uORFs with conserved amino acid sequences (see Methods). Comparison by uORF-Finder of a corrected set of 34000 full-length cDNA sequences from Arabidopsis with a similar set from rice resulted in the identification of conserved peptide uORFs in 44 Arabidopsis genes and 36 rice genes, which together comprise 19 homology groups based on uORF amino acid similarity (Tables 1, 2, 3; Figures 1, 2, 3, 4, 5). All three of the homology groups that had been previously reported were identified by uORF-Finder [1,4]. The other 16 conserved uORFs have not been reported previously. Homologs of these 19 conserved uORF groups also exist in other angiosperm species (Figures 1, 2, 3, 4, 5).

**Table 1:** uORF homology groups and associated mORF molecular function and biological role

Homology group	mORF: known or probable molecular function/domain	Known or inferred biological process	Source
uORF conserved in Arabidopsis and rice			
1	bZIP transcription factor	Sucrose regulation	[89]
2	bHLH transcription factor	Transcriptional control	[68]
3	AdoMetDC	Polyamine biosynthesis: developmental regulation	[1]
4	Unknown; plant-specific	Unknown	BLAST analysis
5	Ankyrin repeat protein	Unknown	Protein domain analysis*
6	Amine oxidase	Unknown	Protein domain analysis*
7	Putative translation initiation factor eIF5	Start codon selection	Protein domain analysis*
8	Similar to Mic-1	Unknown	BLAST analysis
9	Unknown, cysteine-rich	Unknown (Possible novel zinc finger?)	CX_4–7_CX_10_CX_2_HX_5 _tandem repeats
10	MAP kinase	Signal transduction	PlantsP database
11	Trehalose-6-phosphate phosphatase	Trehalose metabolism: developmental regulation	[90]
12	Unknown	Systemically primed response to pathogens	[91]
13	Phosphoethanolamine *N*-methyltransferase	Phosphocholine biosynthesis	[38]
14	HDZip class I transcription factor	Transcriptional control; development	[92,93]
15	bHLH transcription factor	Transcriptional control; responsive to polyamine?	[52,68]
16	MAP kinase	Signal transduction	PlantsP database [99]
17	Unknown	Unknown	
18	Transcription co-activator/repressor HsfB1	Mediator of heat shock response	[94,95]
19	SAUR protein	Mediator of auxin response; calmodulin (CaM) binding	IPR003676; [96]
uORF conserved in Arabidopsis paralogs			
20	Unknown	Unknown	
21	ERF/AP2 transcription factor	Putative regulator of pathogen resistance	[97,98]
22	Unknown	Unknown	
23	MAP kinase	Signal transduction	PlantsP database [99]
24	Unknown	Unknown	
25	Calcium response protein kinase	Ca++/CaM-dependent signal transduction	PlantsP database [100]
26	RING finger (C3HC4-type zinc finger)	Ubiquitination; mediator of protein degradation	Protein domain analysis*

bZIP, basic leucine zipper; bHLH, basic helix-loop-helix; AdoMetDC, S-Adenosylmethionine decarboxylase; Mic-1, colon cancer-associated protein macrophage-inhibitory cytokine 1; MAP kinase, mitogen activated protein kinase; HDZip, homeodomain leucine zipper; ERF/AP2, ethylene response factor/apetala2.

*As determined by InterProScan and NCBI conserved domains search.

**Table 2:** Arabidopsis loci with conserved peptide uORFs identified from Arabidopsis-rice comparison

Homology group	Locus	Gene Name	mORF description	Gene ontology molecular function	Recent duplicate
1	At2g18160.1	*GBF5, AtbZIP2*	Basic leucine zipper (bZIP)	Transcription factor	At4g34590
1	At4g34590.1	*GBF6, ATB2, AtbZIP11*	bZIP	Transcription factor	At2g18160
1	At3g62420.1^a^	*AtbZIP53*	bZIP	Transcription factor	Not found
1	At5g49450.1	*AtbZIP1*	bZIP	Transcription factor	Not found
1	At1g75390.1	*AtbZIP44*	bZIP	Transcription factor	Not found
2	At2g27230.1	*AtBHLH156*^b^	Basic helix-loop-helix (bHLH)	Transcription factor	Not found
2	At2g31280.1	*AtBHLH155*^b^	bHLH	Transcription factor	At1g06150
2	At1g06150.1		bHLH	Transcription factor	At2g31280
3	At3g02470.1	*AdoMetDC1*	AdoMetDC	AdoMetDC	At5g15950
3	At5g15950.1	*AdoMetDC2*	AdoMetDC	AdoMetDC	At3g02470
3	At3g25570.1	*AdoMetDC3*	AdoMetDC	AdoMetDC	Not found
4	At4g25670.1		Expressed transcript	Unknown	At5g52550
4	At4g25690.1		Expressed transcript	Unknown	At5g52550^c^
4	At5g52550.1		Expressed transcript	Unknown	At4g25670
5	At5g61230.1		Ankyrin repeat	Protein binding	At5g07840
5	At5g07840.1		Ankyrin repeat	Protein binding	At5g61230
6	At2g43020.1		Amine oxidase	Oxidoreductase	At3g59050
6	At3g59050.1		Amine oxidase	Oxidoreductase	At2g43020
7	At1g36730.1		Putative eIF-5	Translation initiation factor	Not found
8	At3g12010.1^a^		Similar to Mic-1	Unknown	Not found
9	At5g09670.1 and .2		Expressed transcript	Unknown	At5g64550
9	At5g64550.1		Expressed transcript	Unknown	At5g09670
9	At1g64140.1		Expressed transcript	Unknown	Not found
10	At5g45430.1	*AtMPK23*^d^	MAP kinase, PPC family 4.5.1	ATP binding, protein kinase	At4g19110^e^
10	At4g19110.1	*AtMPK22*^d^	MAP kinase, PPC family 4.5.1	ATP binding, protein kinase	At5g45430^e^
11	At4g12430.1		TPPase	Catalytic activity	At4g22590
11	At4g22590.1		TPPase	Catalytic activity	At4g12430
12	At1g70780.1		Expressed transcript	Unknown	At1g23150
12	At1g23150.1		Expressed transcript	Unknown	At1g70780
13	At3g18000.1	*XPL1, NMT1, PEAMT1*	Phosphoethanolamine *N*-methyltransferase	Methyltransferase	At1g48600
13	At1g48600.2	*NMT2*	Methyltransferase	Methyltransferase	At3g18000
13	At1g73600.1	*NMT3*	Methyltransferase	Methyltransferase	Not found
14	At3g01470.1	*HAT5, HB-1, HD-ZIP-1, ATHB1*	Homeobox	DNA binding, transcription factor, transcriptional activator	Not found
15	At1g29950.2	*AtBHLH144*^b^	bHLH	Transcription factor	Not found
15	At5g50010.1	*AtBHLH145*^b^	bHLH	Transcription factor	Not found
15	At5g64340.1	*AtBHLH142*^b^, *SAC51*	bHLH	Transcription factor	At5g09460
15	At5g09460.1^a^	*AtBHLH143*^b^	bHLH	Transcription factor	At5g64340
16	At3g51630.1	*ZIK1, WNK5*	MAP kinase, PPC family 4.1.5	Protein kinase	Not found
17	At1g58120.1		Expressed transcript	Unknown	Not found
17	At3g53400.1		Expressed transcript	Unknown	Not found
17	At5g03190.1		Expressed transcript	Unknown	Not found
17	At5g01710.1		Expressed transcript	Unknown	Not found
18	At4g36990.1	*AT-HSFB1, ATHSF4*	Heat shock factor	Transcription factor	Not found
19	At5g53590.1		SAUR Auxin responsive	Unknown	Not found

AdoMetDC, S-Adenosylmethionine decarboxylase; PPC, PlantsP protein kinase classification; TPPase, Trehalose-6-phosphate phosphatase.

^a^uORF found upstream of annotated mORF-containing locus (within 2 kb).

^b^As designated by Bailey et al [68], nomenclature agreed upon by both Heim et al [69] and Toledo-Ortiz et al [67].

^c^At4g25670 and At4g25690 (tandem duplicates) have the same recent retained duplicate (not reported by Blanc and Wolfe).

^d^As designated by the PlantsP database [99].

^e^Not found in Blanc and Wolfe's initial analysis of ohnologs, but synteny and homology suggest they are retained recent duplicates.

**Table 3:** Rice loci with conserved peptide uORFs identified from Arabidopsis-rice comparison

Homology group	Locus	mORF description	Gene ontology molecular function
1	LOC_Os02g03960	bZIP	DNA binding, transcription factor
1	LOC_Os09g13570	bZIP	DNA binding, transcription factor
1	LOC_Os05g03860	bZIP	DNA binding, transcription factor
1	LOC_Os03g19370	bZIP	DNA binding, transcription factor
1	LOC_Os12g37410	bZIP	DNA binding, transcription factor
2	LOC_Os12g06330	bHLH	Transcription factor
3	LOC_Os02g39790	AdoMetDC	AdoMetDC activity
3	LOC_Os04g42090	AdoMetDC	AdoMetDC activity
3	LOC_Os09g25620	AdoMetDC	AdoMetDC activity
4	LOC_Os02g01360	Expressed transcript	Unknown
5	LOC_Os02g01240, 133165–133284*	Ankyrin repeat	Protein binding, Acyl CoA binding
6	LOC_Os04g53190, 31234580–31234757*	Amine oxidase	Amine oxidase
7	LOC_Os09g15770	IF2B and IF5 domains	Translation initiation
7	LOC_Os06g48350	IF2B and IF5 domains	Translation initiation
8	LOC_Os10g26140	Similar to Mic-1	Unknown
9	LOC_Os04g38520	Expressed transcript	Transcription factor
9	LOC_Os02g36590, 22043438–22043536*	Expressed transcript	Transcription factor
9	LOC_Os01g43370	Expressed transcript	Transcription factor
9	LOC_Os02g15880, 8987945–8988028*	Expressed transcript	Transcription factor
10	LOC_Os06g02550	Protein kinase	Kinase activity
10	LOC_Os02g47220, 28767408–28767530*	Protein kinase	Kinase activity
11	LOC_Os02g44230	TPPase	Trehalose phosphatase
11	LOC_Os10g40550	TPPase	Trehalose phosphatase
12	LOC_Os02g21920	Expressed transcript	Unknown
13	LOC_Os01g50030	Methyltransferase	Phosphoethanolamine *N*-methyltransferase activity
13	LOC_Os05g47540	Methyltransferase	Phosphoethanolamine *N*-methyltransferase activity
14	LOC_Os08g32080, 19755174–19755260*	Homeobox	DNA binding, transcription factor, protein binding
15	LOC_Os02g21090	bHLH	Transcription factor
15	LOC_Os01g43680, 25011025–25012089*	bHLH	Transcription factor
15	LOC_Os03g39432, 21870203–21870427* (LOC_Os03g39432 v.4 TIGR annotation)	bHLH	Transcription factor
15	LOC_Os03g27390	bHLH	Unknown
16	LOC_Os11g02300	Protein kinase	Protein kinase
17	LOC_Os07g42830, 25650516–25650623* (LOC_Os0742834 v.4 TIGR annotation)	Expressed transcript	Unknown
17	LOC_Os02g52300	Expressed transcript	Unknown
18	LOC_Os09g28350 (LOC_Os09g28354 v.4 TIGR annotation)	Heat shock factor	DNA binding, transcription factor
19	LOC_Os10g36700 (LOC_Os10g36699 v.4 TIGR annotation)	Auxin responsive	Unknown

All locus identifiers based on version 3 TIGR pseudomolecule assembly except where noted. AdoMetDC, S-Adenosylmethionine decarboxylase; TPPase, Trehalose-6-phosphate phosphatase.

*Locus numbers indicate mORF location, and coordinates indicate uORF location in intergenic region on the same chromosome.

### Comparison of Arabidopsis homologs detects additional conserved uORFs

**Figure 6:**
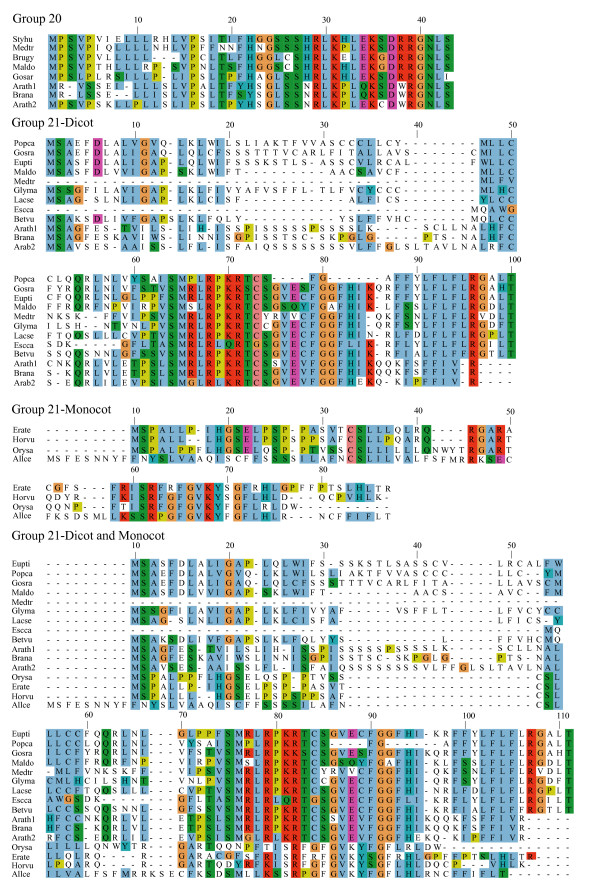
**Alignments of plant uORF homology groups 20 and 21**. Details as in Figure 1. Groups with similarity in both the monocot and dicot lineages are shown as separate alignments and as a joint alignment.

**Figure 7:**
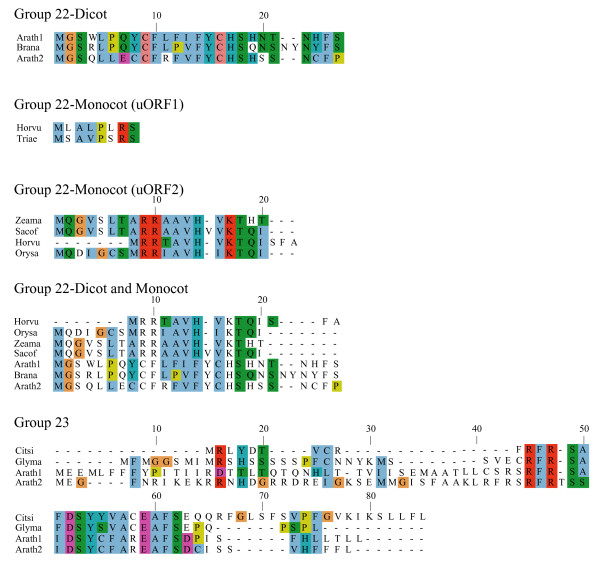
**Alignments of plant uORF homology groups 22 and 23**. Details as in Figure 6.

**Figure 8:**
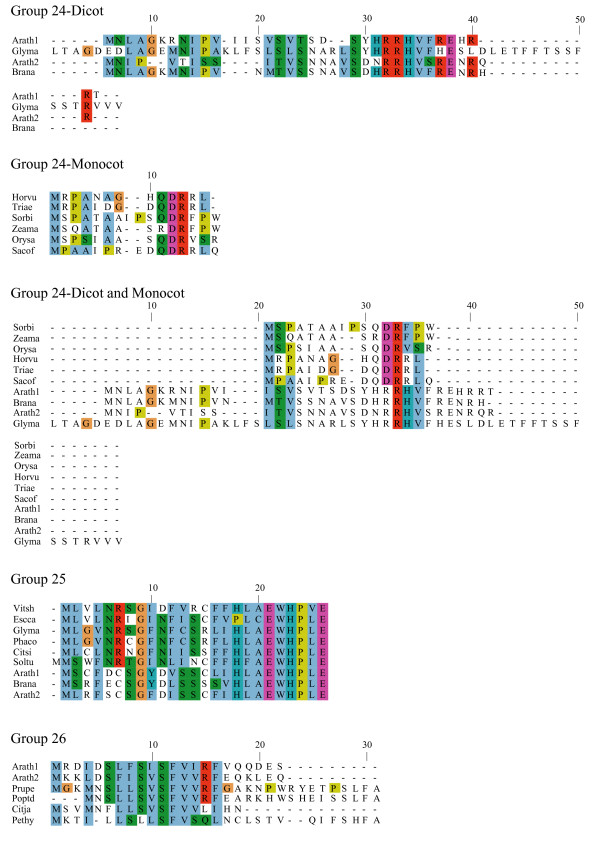
**Alignments of plant uORF homology groups 24–26**. Details as in Figure 6.

Conserved uORFs that are not sufficiently well conserved to be detected in a rice-Arabidopsis comparison could conceivably be detected in ohnologs, homologous genes arising by whole-genome duplication (WGD) [30], and in paralogs, homologous genes arising from segmental duplication or tandem duplication. Modification of uORF-Finder allowed comparison of each full-length cDNA to all other cDNAs in the same collection (see Methods), and identified seven additional conserved uORF homology groups (Tables 1 and 4; Figures 6, 7, 8). Six of these pairs are ohnologs, created by the most recent WGD (24–40 Mya) in an ancestor of Arabidopsis [31-33]. The seventh pair is not found in syntenic regions and is most likely a paralogous pair. It appears to have arisen at about the same time as the recent WGD event because its synonymous substitution frequency (*K*_*s *_value) of 0.7 is similar to the median *K*_*s *_of recent duplicate pairs (0.8) and is within their *K*_*s *_range (0.4–1.6) [32]. The corresponding rice genes in four of the seven homology groups possess uORFs, but lack sufficient uORF sequence similarity to have been detected in the Arabidopsis-rice comparison (Figures 6, 7, 8).

### Purifying selection maintains uORF amino acid sequences

Pairwise *K*_*a*_/*K*_*s *_tests for selection on amino acid sequences were applied to each uORF homology group and their associated mORFs to determine whether uORF amino acid sequences are under selective constraints similar to their associated mORFs. Both an approximate method (Yn00) and a maximum likelihood method (codeml) were used to calculate mean pairwise *K*_*a*_/*K*_*s *_ratios for each group. A *K*_*a*_/*K*_*s *_ratio less than 1 implies that negative, or purifying, selection has acted on the sequence, a ratio equal to 1 suggests drift, and a ratio greater than 1 indicates that positive selection has acted on an amino acid sequence. It is also true that conservation at the nucleotide level, not the amino acid level, can drive the *K*_*a*_/*K*_*s *_ratio to one. Analysis of all 26 homology groups showed that generally both uORFs and mORFs have been under mild to strong purifying selection since the divergence of each gene pair (Table 5) and these low *K*_*a*_/*K*_*s *_ratios suggest that the conservation is at the amino acid level, not simply at the nucleotide level.

**Table 4:** Arabidopsis loci with conserved peptide uORFs identified from Arabidopsis-Arabidopsis comparison

Homology group	Locus	Gene name	mORF description	Gene ontology molecular function	Recent duplicate
20	At3g53670.1		Expressed transcript	Unknown	At2g37480^a^
20	At2g37480.1		Expressed transcript	Unknown	At3g53670^a^
21	At1g68550.1	*AtERF#118*^b^	Group VI-L ERF/AP2 transcription factor	Transcription factor	At1g25470
21	At1g25470.1	*AtERF#116*^b^	Group VI-L ERF/AP2 transcription factor	Transcription factor	At1g68550
22	At1g16860.1		Expressed transcript	Unknown	At1g78880
22	At1g78880.1		Expressed transcript	Unknown	At1g16860
23	At1g64630.1	*ZIK10*	MAP kinase, PPC Family 4.1.5	Transcription factor	Not found
23	At5g41990.1	*WNK8*/*ZIK6*	MAP kinase, PPC family 4.1.5	Protein kinase	Not found
24	At3g22970.1		Expressed transcript	Unknown	At4g14620
24	At4g14620.1		Expressed transcript	Unknown	At3g22970
25	At3g45240.1^c^		Calcium response kinase, PPC family 4.2.7	ATP binding, protein kinase	At5g60550
25	At5g60550.1		Calcium response kinase, PPC family 4.2.7	ATP binding, protein kinase	At3g45240
26	At3g10910.1		Zinc finger, C3HC4-type (RING finger)	Protein binding, zinc ion binding	At5g05280
26	At5g05280.1		Zinc finger, C3HC4-type (RING finger)	Protein binding, zinc ion binding	At3g10910

ERF/AP2, Ethylene Response Factor/Apetela 2 transcription factor; PPC, PlantsP protein kinase classification.

^a^Blanc and Wolfe (2004) report that At2g3790 and At3g53670 are retained recent duplicates, but the At2g3790 locus has since been replaced by At2g3780.

^b^As defined by Nakano, et al [97] and previously characterized as part of subfamily B-6 by Sakuma, et al [100].

^c^uORF found upstream of annotated mORF-containing locus (within 2 kb).

**Table 5:** Mean pairwise *K*_*a*_/*K*_*s *_values for all pairwise combinations of a given homology group using two methods (yn00 and codeml).

Homology group	uORF		mORF	
	
	yn00	codeml	yn00	codeml
1	0.20	0.16	0.22	0.11
2	0.28	0.15	0.29	0.19
3	0.13	0.11	0.15	0.09
4	0.19	0.18	0.21	0.22
5	0.06	0.06	0.06	0.08
6	0.43	0.01^a^	0.10	0.08
7	0.43	0.89	0.09	0.05
8	0.14	0.01^a^	0.11	0.09
9	0.19	0.05	0.20	0.09
10.1^b^	0.69	0.48	0.10	0.10
10.2^b^	0.70	0.64		
11	0.13	0.09	0.13	0.09
12	0.25	0.26	0.15	0.09
13	0.07	0.04	0.10	0.09
14	0.17	0.05	0.14	0.01^a^
15.1^b^	0.31	0.17	0.34	0.21
15.2^b^	0.03	0.07		
15.3^b^	0.37	0.16		
16	0.30	0.11	0.10	0.11
17	0.28	0.24	0.41	0.11
18	0.26	0.01^a^	0.15	0.01^a^
19	0.00^a^	0.01^a^	0.01^a^	0.01^a^

20	0.13	0.17	0.48	0.39
21	0.47	0.44	0.11	0.09
22	0.52	0.16	0.09	0.09
23	0.57	0.43	0.23	0.21
24	0.18	0.20	0.22	0.20
25	0.53	0.50	0.16	0.14
26	0.37	0.28	0.23	0.22

^a^*K*_*a *_or *K*_*s *_values too high to determine *K*_*a*_/*K*_*s *_ratio accurately.

^b^Decimal points after homology group numbers are used when multiple independent uORF peptides are conserved within a single transcript.

One possible explanation for low *K*_*a*_/*K*_*s *_ratios in the putative uORFs invokes an incomplete splicing of the full-length cDNAs for which the uORF and mORF are normally fused. To address this possibility, all Genbank Arabidopsis ESTs were screened for evidence of uORF-mORF translational fusions. No ORFs were found to run continuously between the uORF and mORF, with one exception. A fusion product (Genbank accession no. DR353698) was identified between the N-terminal and central region of the uORF and the central and C-terminal region of the mORF found at locus At5g03190 (group 17). Classification of this putative uORF is shown in Table 1 for two reasons. Firstly, the four uORF C-terminal amino acids that are excluded in the fusion EST are perfectly conserved in monocot and dicot members, and the position of their stop codon is perfectly conserved, therefore it is difficult to explain this conservation if the uORF is not translated. Secondly, the N-terminal portion of the mORF that is removed in the fusion EST is similar between three Arabidopsis loci of the same homology group, with the start codon position also being conserved in these three members. It is likely, therefore, that the fusion EST represents an alternatively spliced form of this transcript, but further characterization of this locus will be needed to support this conclusion. Most of the homology groups show uORFs with conserved amino acid residues at the C-terminus and an identical positioning of the uORF stop codon (Figures 1, 2, 3, 4, 5, 6, 7, 8). This would suggest that the full-length cDNAs are fully spliced and are not erroneously predicting uORF sequences due to incomplete splicing.

### Conserved features of uORF sequences

**Figure 9:**
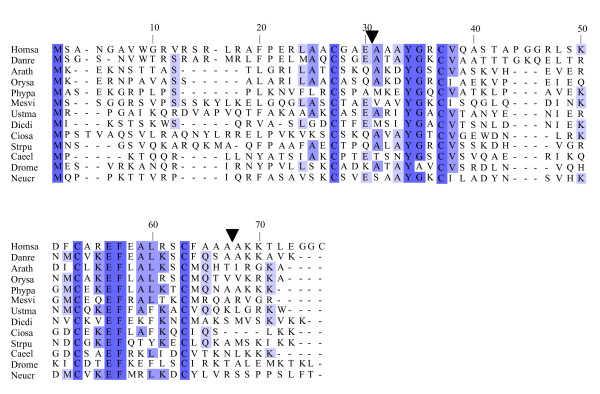
**Group 8 small ORF/uORF alignment and percent identity across various eukaryotes**. Representative eukaryotic species aligned using Muscle and displayed by percent identity using Jalview. Arrowheads represent two conserved intron positions for all but Mesvi (no genomic support), Dicdi (first but not second intron present), Ciosa (no introns), Caeel (no introns), Drome (no introns), and Neucr (first but not second intron present based on predicted mRNA). See main text for abbreviated species names and Genbank accession number, cDNA clone number, or genome identifier.

**Figure 10:**
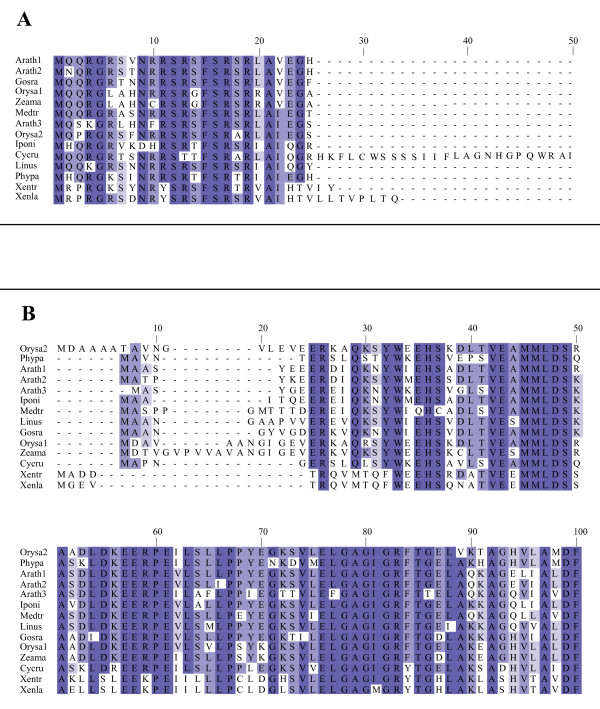
**Group 13 alignment and percent identity of (A) uORF and (B) mORF sequences**. Representative eukaryotic species were aligned using Muscle and displayed using Jalview. Panel A alignment is restricted to the first 50 amino acid positions, which excludes the full 92 amino acid uORF of *Cycas rumphii*. All other uORFs are shown in their entirety. Panel B alignment is restricted to the first 100 amino acid positions of the mORFs. See main text for abbreviated species names and Genbank accession number, cDNA clone number, or genome identifier.

The lengths of uORFs vary to differing degrees within and among homology groups, but in amino acid sequence alignments nearly all groups exhibit considerable conservation of the position of the N-terminus and/or the C-terminus, i.e., length variation is usually due to a variable region in the middle or at one end of the uORF (Table 6; Figures 1, 2, 3, 4, 5, 6, 7, 8). The amino acid sequences of some uORFs possess potentially interesting features. Notably, some uORF groups possess regions rich in serine, threonine, and/or tyrosine, and others possess regions rich in lysine and/or arginine. Two homology groups are particularly noteworthy: Group 8 uORFs specify peptides with a coiled coil-helix, coiled coil-helix (CHCH) domain (Pfam accession number PF06747; Figure 9), and group 13 uORFs encode peptides that are extremely serine/arginine-rich (Figure 10). Both of these unusual peptides will be discussed in further detail below.

**Table 6:** uORF features conserved between Arabidopsis and rice

uORF homology group	uORF length (amino acids)	Conserved sequence features				
		
		Length conserved at N- and C-Termini	N-terminus (amino acids)	Middle (amino acids)	C-terminus (amino acids)	Overall
uORF features conserved between Arabidopsis and rice						
1	25–43	C-terminus			SY-rich: 5/14	20–39% STY
2	34–39	C-terminus			KR-rich: 5–6/20	18–24% KR
3	50–54	N- and C-termini	K-rich: 4/9	S-rich: 5–6/6	KR-rich: 4–5/16, SY-rich: 4–5/12	17–23% KR. 22–29% SY
4	52–55	N- and C-termini			SY-rich: 7/30	21–23% STY
5	38–41	N- and C-termini				
6	55–68	N-terminus				
7	57–105	N-terminus	STY-rich: 6–7/22	KR-rich: 4/5–8		
8	61–62	N- and C-termini				CHCH domain, 17% KR
9	17–33	N-terminus				
10.1	41	N- and C-termini				
11	24–44	C-terminus			KR-rich: 5–6/15	25% KR
12	39–51	N- and C-termini				
13	25	N- and C-termini		RS-rich: 10–12/18		40–48% RS
14	29	N-terminus		ST-rich: 4–6/10		14–32% STY
15.1	18–27	N- and C-termini			8/9 hydrophobic	
15.3	43–54	N- and C-termini			13/14 completely conserved	
16	40–62	C-terminus				
17	36–45	C-terminus				
18	36–38	Neither				
19	30–34	N-terminus				29% ST
uORF features conserved between Arabidopsis paralogs						
20	41–43	N- and C-termini	STY-rich: 8–9/27			23% STY
21	87–90	N- and C-termini		ST-rich: 11–12/17–22		22–25% ST
22	25	N- and C-termini				
23	69–71	Neither				
24	31–34	N- and C-termini				
25	25	N- and C-termini				
26	22	N-terminus				

### Most genes with conserved uORFs appear to have regulatory functions

A total of 31% of mORFs encoded by conserved peptide uORF loci in Arabidopsis were predicted to be a transcription factor, as determined by GO molecular function terms (Tables 2 and 4), whereas only 5.9% of all Arabidopsis loci are predicted to encode transcription factors [34]. Thus, genes predicted to encode transcription factors are significantly overrepresented (p = 1.2 × 10^-7^) among conserved peptide uORF loci. In each case, GO terms were validated by manual annotation of protein functions using domain predictions from NCBI Conserved Domain and InterProScan Database searches [35,36]. A variety of different types of transcription factors, including bZIP, Ethylene Response Factor/Apetala 2-like (ERF/AP2-like), basic helix-loop-helix (bHLH), and homeobox proteins, are represented among conserved peptide uORF loci with no demonstrable bias. No other GO terms were found to be significantly over- or under-represented in the uORF data set.

Biological functions could be inferred for 16 of the 26 uORF homology groups (Table 1). Six groups encode transcription factor homologs and so are presumably involved in transcriptional control (1, 2, 14, 15, 18, and 21). Five groups are likely to be involved in signal transduction, including four protein kinases and a putative calmodulin-binding protein involved in auxin response (groups 10, 16, 19, 23, 25). Two groups are involved in the metabolism of small molecules that regulate plant development: polyamines (group 3) [1] and trehalose (group 11) [37]. One group (13) encodes the key enzyme in the biosynthesis of phosphocholine, which is an intermediate in biosynthesis of phosphatidylcholine and phosphatidic acid; phosphocholine levels influence levels of phosphatidic acid, an important physiological and developmental signal molecule [38-40]. Group 7 putatively encodes translation initiation factor eIF5, which influences start codon selection, and Group 26 encodes a RING finger protein, suggesting a role in targeted protein turnover by ubiquitination. Of the remaining 10 groups, 8 encode predicted proteins of unknown function, 1 encodes an ankyrin-repeat protein, and 1 encodes an amine oxidase. Thus, all but two families of conserved uORF genes whose functions are known or can be inferred potentially play a regulatory role in the biology of plants.

### Genes with conserved uORFs were preferentially retained after whole genome duplication

Since the most recent WGD event in the Arabidopsis lineage, only 14% of the original gene pairs present in the ancestral tetraploid have been retained as a duplicate pair in the extant Arabidopsis genome, i.e., for the remaining 86% of ancestral gene pairs, one member has been lost [32]. Among 31 ancestral gene pairs that possessed conserved uORFs at the time immediately following the genome duplication, 12 (39%) pairs have been retained in the present Arabidopsis genome (Table 2), which is significantly higher than the genome-wide average (p = 0.0005). The conserved uORF was retained in both copies of each of the twelve retained duplicate pairs. Retention of these 12 uORFs in both paralogs suggests that they act *in cis*, consistent with the expectation that uORFs typically control translation of downstream mORFs on the same RNA molecule [41].

The overrepresentation of transcription factors among conserved uORF loci could be due, in part, to preferential retention of transcription factor recent duplicates (22.7% retention of transcription factor duplicates vs 14.4% retention genome-wide) [32], but this alone does not account for the high frequency of predicted transcription factors among the uORF loci. When duplicate history bias is removed by calculating GO term frequencies of the pre-genome-duplication set of loci, transcription factors are still overrepresented (11/31 loci, or 35%).

### Conserved angiosperm uORF peptide sequences in primitive plants and other eukaryotes

To determine whether any of the 19 uORF homology groups conserved between rice and Arabidopsis might also be present in other eukaryotes, we searched for uORF sequences in all Genbank eukaryotic ESTs. Amino acid sequences similar to four homology groups (3, 8, 13, and 15) were detected in non-angiosperms. Group 15 was found only as distantly as a fern (*Adiantum*); group 3 was found as far from angiosperms as the green algae (*Ulva*); group 13 was found in an animal (*Xenopus tropicalis*); and group 8 uORF sequence was found in primitive plants, animals, fungi, and a slime mold (Figures 9 and 10). Another algal sequence (*Chlamydomonas*) from the Genbank non-redundant database was identified belonging to group 3 (Genbank: AJ841703). The group 13 uORF homolog found in a *X. tropicalis *EST was also found in a genomic contig sequence [42] in which the uORF homolog is flanked by genes that are more similar to animal sequences than to any known plant sequences. Thus, this group 13 uORF homolog most likely exists in the *Xenopus *genome rather than being an EST library contaminant.

**Figure 11:**
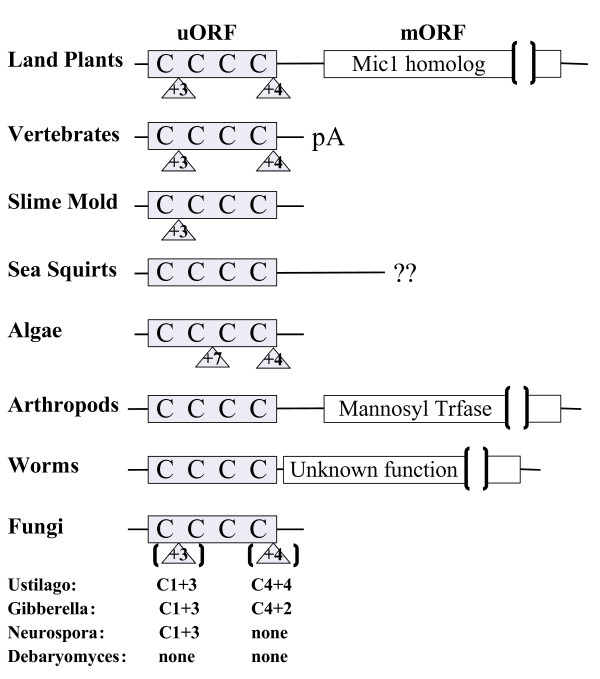
**Diagrammatic representation of Group 8 features among eukaryotes**. Light grey boxes represent small ORFs/uORFs, four perfectly conserved cysteine residues are shown as 'C', and numbers within triangles represent the number of amino acids between the immediately preceding cysteine and an intron. Brackets surrounding fungal introns represent the variable nature of the intron position and/or presence. White boxes show mORFs directly downstream of the uORFs in a given lineage. Presence of a polyA tail is likely to occur in vertebrates (pA; see Results). Question marks indicate mORFs could be present, but insufficient EST sequence is available to infer this feature reliably.

Sequences similar to group 8 Arabidopsis and rice uORFs were found in most eukaryotes, but transcript sequence following the uORF varied among the different lineages. All land plant uORFs were associated with macrophage inhibitory cytokine-1-like (Mic1-like) mORF sequences while the mORFs downstream of the group 8 uORF homologs in nematodes and arthropods code for an unknown protein and a putative mannosyl transferase, respectively (Figure 11). Available EST sequences for each of the group 8 uORF homologs in mammals, fungi, algae, and slime mold end shortly after the conserved peptide uORF, suggesting that in these eukaryotes the uORF homolog is not associated with a mORF and is simply a short ORF. This is further supported by more than 10 human ESTs that end at the same position and include a polyA sequence. In the sea squirt lineage a putative mORF is present in the EST sequences, but a full-length cDNA sequence will be needed to further investigate this possibility.

Although there is variability in the sequences found downstream of group 8 uORFs, three features of these uORF homologs are relatively well conserved: the length of the predicted uORF, the relative positions of four cysteine codons, and the positions of two introns (Figure 9). The length of the uORF peptide ranges from 51 amino acids in *Haemonchus *(nematode), to 74 amino acids in humans, and length is even more highly conserved within each of the land plant, arthropod, nematode, fungal, and vertebrate lineages (59–62, 65–69, 51–68, 54–66, and 69–74 amino acids, respectively). Four cysteine residues consistently align in all eukaryotes, with nine amino acids separating the first and second cysteine residues, as well as the third and fourth cysteine residues, whereas 11–15 residues separate the second and third cysteines. Two intron positions are perfectly conserved among the land plants, vertebrates, and at least one member of the fungal lineage. The first intron lies between the third and fourth amino acids following the first conserved cysteine position, and the second intron lies between the fourth and fifth amino acids following the fourth conserved cysteine position (Figure 11). The first and/or second intron positions are present in *Dictyostelium*, algae, and some fungi, but are absent in nematodes, arthropods, and sea squirts.

The four cysteines are part of a putative coiled coil-helix, coiled coil-helix (CHCH) domain (Pfam accession number PF06747), also found in three small yeast proteins, Cox17p, Cox19p, and Mrp10p. Cox17p and Cox19p are required for assembly of functional cytochrome oxidase and Mrp10p is homologous to a nuclear-encoded mitochondrial ribosomal protein. A hypothetical human gene, CHCH domain 7 (*CHCHD7*), is also similar to the group 8 uORF, as determined by BLAST similarity searches.

### Phylogenetic relationships among group 8-like ORFs

**Figure 12:**
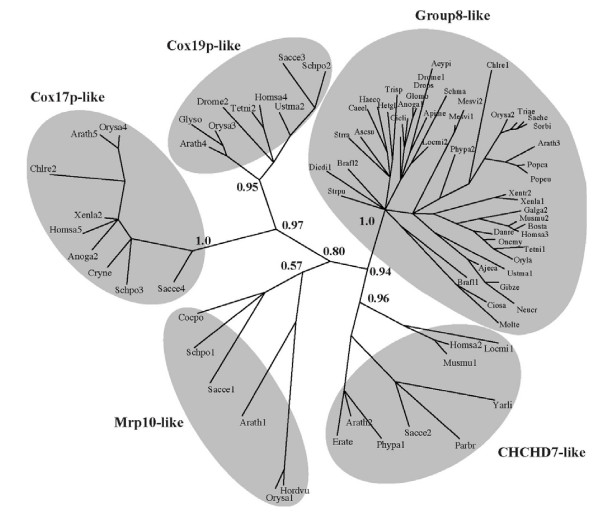
**Phylogenetic tree depicting CHCH domain-containing genes and alignment**. Unrooted phylogenetic tree generated using MrBayes 3.0. See main text for abbreviated species names and Genbank accession number, cDNA clone number, or genome identifier.

Fungal, animal, and plant representatives of each CHCH-containing ORF were identified using a BLAST search, and their evolutionary relationships were inferred using a Bayesian phylogenetic analysis (Figure 12; Additional file 1). Animal Mrp10p-like (Genbank: BC075310, DR155443 and BX935835), Debaryomyces group 8-like (Genbank: NC_006045), and Dictyostelium Cox19p-like (Genbank: XM_631387) sequences were more divergent than other sequences, causing long branch attraction [43]. Thus, these sequences were removed from the analysis to prevent tree topology distortion. Five distinct clades were observed, which we refer to as Cox17p-like, Cox19p-like, Mrp10p-like, CHCHD7-like, and uORF group 8-like (Figure 12). All clades but one (Mrp10p-like) contain representatives from fungi, animals, and plants and are strongly supported, showing branch order probabilities greater than 0.8, which suggests that these sequences emerged in a common eukaryotic ancestor and have since diverged in the three lineages. Mrp10p-like sequences do not strongly group independently of other branches (P = 0.57), which could be due to highly divergent amino acid sequence represented by relatively long branches. The tree shows that the group 8-like proteins are a distinct clade from other CHCH domain proteins (P = 1.0), and that CHCHD7-like proteins are more closely related to group 8-like members than to other CHCH-containing proteins (P = 0.94). The tree topology also indicates that Cox17p-like and Cox19p-like genes are more closely related to each other than to other CHCH proteins (P = 0.97).

**Figure 13:**
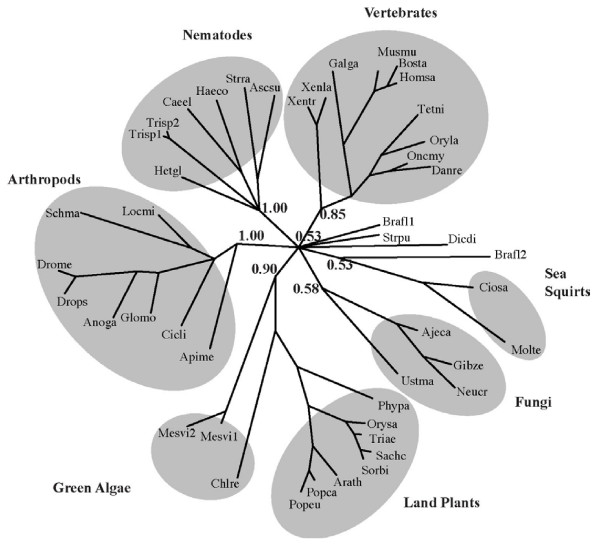
**Phylogenetic tree depicting group 8 small ORFs/uORFs and alignment**. Unrooted phylogenetic tree generated using MrBayes 3.0. See main text for abbreviated species names and Genbank accession number, cDNA clone number, or genome identifier.

A separate phylogenetic analysis of the 46 group 8-like sequences shows that most cluster into five taxonomic groups (plants and green algae, arthropods, nematodes, vertebrates, and fungi) with strong branch support (0.85–1.00) in all but the fungal lineage (0.58; Figure 13). Sea squirt sequences group with one of two *Branchiostoma *sequences with weak branch support (0.53). *Dictyostelium*, sea urchin (*Strongylocentrotus*), and one further *Branchiostoma *sequence do not group with any of these with weak support (0.53). Sea squirt, *Branchiostoma*, and sea urchin sequences should be more similar to other deuterastomes (includes the vertebrate lineage) than other organisms, but the short group 8-like sequence alignment could prevent resolution of correct evolutionary relationships of some groups (Additional file 2). Despite weakly supported branches, there is strong support for independent clustering of the arthropods, nematodes, vertebrates and plants, as expected.

Although two *Branchiostoma *group 8-like sequences (Brafl1 and 2) suggest that there has been a duplication event within this lineage, there is no evidence for maintenance of ancient group 8-like gene duplications occurring within the plant, vertebrate, nematode, arthropod, or fungal lineages. In Arabidopsis both the recent and ancient duplicates from two WGD events have been lost from the genome. Only the *Mesostigma *genome contains two group 8-like transcripts. Their short branch lengths indicate that this duplication occurred relatively recently and it is possible that insufficient time has passed for loss of the second copy.

## Discussion and conclusion

Comparative analysis by uORF-Finder of 5' UTRs in full-length cDNAs from two distantly related plant species, rice and Arabidopsis, identified conserved peptide uORFs in 58 Arabidopsis loci that comprised 26 uORF homology groups and in 36 rice loci that comprised 19 homology groups, increasing the number of known conserved uORF homology groups from two to 26 and providing useful, new information for investigations of regulatory biology. Because full-length cDNAs derived from both Arabidopsis and rice only represent a fraction of all nuclear genes, not all conserved uORFs are expected to be detected by this approach. Extrapolation to the whole Arabidopsis genome suggests that it possesses approximately 61 to 102 genes with conserved peptide uORFs that are also conserved in the rice genome (see Methods for calculation). An additional 24 conserved peptide uORF genes are predicted among Arabidopsis loci with retained duplicates from the most recent WGD event. In all, there are likely to be approximately 99–140 genes, or 0.38–0.53% of all protein-coding genes, with conserved peptide uORFs in the Arabidopsis genome. Because short conserved uORFs (<20 amino acids) would not have been detected by uORF-Finder, this is a conservative estimate.

To find additional conserved uORFs, more extensive collections of full-length cDNA sequences will need to be developed and/or 5' UTRs predicted from genomic sequence will be required. As full-length cDNA sequence resources become available for other plant species, such as maize [44] and poplar [45], it should be possible to identify additional conserved uORFs that might be specific to taxonomic groups, such as monocotyledons or dicotyledons. Similarly, analysis of ancient tetraploidy events in species such as poplar and maize might be able to identify uORFs conserved between retained duplicates.

### Conserved uORF genes are regulatory genes

Based on the study of a few hundred genes, it has been suggested that uORFs are usually associated with mORFs that encode proteins that regulate cell growth [41,46], but a genome-wide study of upstream AUGs (uAUGs) found no correlation of uAUG-containing transcripts with any particular gene ontology (GO) molecular function term in mammalian transcripts [6]. These observations did not differentiate between sequence-dependent and sequence-independent uORFs. Our analysis shows that genes encoding transcription factors are overrepresented among genes predicted to encode conserved peptide uORFs, representing almost one third of the 58 Arabidopsis loci as compared to 6% of all genes. Moreover, nearly all genes whose function can be reasonably inferred appear to play some regulatory role in the biology of plants.

### Do conserved peptide uORFs mediate feedback translational regulation by small regulatory molecules?

Certain eukaryotic conserved peptide uORFs are known to control translation of a downstream mORF in response to a metabolic product such as arginine or polyamines [4,14,47]. In the case of the fungal arginine-regulated carbamoyl-phosphate synthase subunit, a uORF codes for the arginine attenuator peptide that responds to increased arginine concentrations by causing ribosomes to stall near the 3' end of the uORF, interfering with ribosome scanning and translation of the downstream mORF [14]. A similar mechanism has been elucidated for the regulation of AdoMetDC in which the uORF peptide interferes with the termination of uORF translation in a polyamine-dependent manner [48,49]. In plants, sucrose is a signaling molecule that controls not only the transcription of many genes, but also translation of a class of bZIP transcription factors via their conserved uORF, suggesting the possibility of sucrose interaction with a uORF-encoded peptide to regulate translation downstream [4].

Our analysis identified not only these previously known examples of genes involved in pathways exhibiting small molecule feedback in a uORF sequence-dependent manner, but several additional genes that might also act via this mechanism. One is the conserved group 13 uORFs, which are present in genes that encode phosphoethanolamine *N*-methyltransferase (PEAMT/NMT), the key enzyme in phosphocholine (PCho) biosynthesis. Recently, *NMT1 *has been shown to contain a uORF that differentially affects translation of the mORF in response to exogenously added choline [50]. This effect is observed when the uORF start codon is abolished but it remains to be determined whether the response to choline is uORF sequence-specific. Intriguingly, the group 13 uORF peptide is rich in arginine and serine (40–48% in Arabidopsis and rice genes; Table 6). A variety of arginine-rich peptides 15–20 amino acids long with 5 or more arginines bind to specific RNA sequences [51]. The predicted group 13 uORF peptide has 5–7 arginines in a 16–17 amino acid region, well within this range, suggesting the possibility that it might bind to a specific RNA sequence, perhaps in *PEAMT*/*NMT *transcripts. The fact that the group 13 uORF peptide was also found in Xenopus suggests that its regulatory role is widespread in eukaryotes.

Another example is homology group 11, whose mORFs are predicted to encode trehalose-6-phosphate phosphatase (TPPase); trehalose-6-phosphate is postulated to regulate sugar metabolism in plants [37]. In summary, sucrose, polyamines, phosphatidic acid, and trehalose-6-phosphate are possible regulators of translation of downstream mORFs through interaction with conserved uORFs. Also interesting in this light are group 19, which specifies an auxin-induced calmodulin-binding homolog, and group 15, which encodes a bHLH transcription factor that is believed to be subject to translational control through its conserved uORF by spermine synthase [52]. Spermine is a polyamine signal molecule necessary for normal plant growth and defense responses.

As mentioned, six conserved uORF families specify transcription factors, one of which is regulated by the small signaling molecule sucrose. In plants, transcription factors often act quantitatively to control target gene expression proportionate to transcription factor concentration [53]. Therefore, it is interesting to consider the possibility that translational control of transcription factor protein levels could be mediated by interaction of a conserved uORF peptide with a metabolite. This might be an effective means for quantitatively modulating the levels of expression of a pathway or network of downstream genes, for instance, in response to changing physiological or environmental conditions. This logic can equally be applied to other key control proteins and their uORFs.

### How is translational control mediated by conserved peptide uORFs?

If conserved uORF peptides can regulate mORF levels in response to small molecules, they are clearly analogous to RNA sensors and riboswitches that sense small molecules and regulate transcript translation accordingly [28,54]. It is interesting to think of conserved peptide uORFs too as sensors of cellular, physiological, or developmental conditions. Although the role of conserved uORFs as 'sensors' of cellular metabolites has been clearly established in the cases of polyamine, sucrose, and arginine concentration, it is still not clear how uORF peptides gauge cellular conditions. uORF peptides could affect mORF translation by interacting directly with the ribosomal complex, by associating with other proteins that influence the translational machinery, and/or by stabilizing or destabilizing RNA secondary structures in the 5' UTR that impede or promote mORF translation. Given the variety of uORF peptides represented in the 26 homology groups, each of these possibilities could occur one or more times.

It is perhaps interesting to note also that the uORFs of 9 homology groups are rich in serine, threonine, and/or tyrosine. These amino acids are potential targets for phosphorylation that conceivably could promote or inhibit ribosome stalling or initiation at downstream mORFs. As mentioned above, lysine/arginine-rich motifs could function in RNA binding [51].

### Effect of nonsense-mediated decay on uORF transcripts

Because uORFs create a premature termination codon (PTC), the nonsense-mediated decay (NMD) system might target uORF transcripts for degradation. Yoine et al [55] carried out a microarray analysis of plants mutant in the *UPF1 *ortholog, which is required for NMD.

Among 75 genes that Yoine et al identified that accumulate transcripts at more than twice the level in the *upf1 *mutant as in wild type Arabidopsis, we found representatives of seven uORF homology groups (1, 7, 10, 12, 13, 15, and 17), suggesting that these uORF transcripts are susceptible to nonsense-mediated decay. The uORFs in these groups might work in a manner analogous to the uORF arginine attenuator protein (AAP) in the fungal CPA1 transcript. The CPA1 transcript exclusively exhibits increased levels of degradation via NMD when the AAP inhibits translation termination in response to high levels of arginine, ultimately decreasing translation using a two-pronged approach [56]. Similarly, the above-identified plant uORFs could intensify translational inhibition of their associated mORFs by both blocking the ribosome physically and inducing the NMD pathway.

### Evolutionary emergence of uORFs and a 'transcriptional fusion' model

Very little is known about how uORFs arise. In the extant rice and Arabidopsis genomes, sequences homologous to uORFs identified by uORF-Finder were observed only in 5' UTRs and never as part of another mORF, within 3' UTRs, within introns, or in non-transcribed regions. Possible origins of 5' UTR ORFs include (a) fragmentation of mORF sequences, (b) creation of an AUG or alternate start codon by random mutation within the 5' UTR and subsequent selection for the peptide sequence, and (c) relocation of other ORF sequences within the genome to the 5' UTR or upstream region of a given gene and subsequent transcriptional fusion of the two ORFs.

Transcriptional fusions occur in an estimated 2% of adjacently transcribed mRNAs in the human genome [57]. The evolutionary history of uORF homology group 8 suggests a stable transcriptional fusion model leading to uORF emergence in plants, arthropods and nematodes. Group 8 uORFs are associated with three independent mORFs in the land plant, arthropod and nematode lineages, while the vertebrate, slime mold, algal, and fungal small ORFs that are orthologous to group 8 uORFs do not seem to be associated with mORFs. Given the phylogenetic relationships among these species [58], the most parsimonious explanation for the evolutionary origin of group 8 uORFs is that they originated as a small ORF transcribed independently of a mORF. Subsequently, this small ORF gene was displaced via genome rearrangements or transposition events to regions upstream of three independent large ORFs resulting in transcriptional fusions of the two previously independent transcripts. The uORFs and mORFs in the plant, nematode, and arthropod lineages have remained associated within the same transcript for 300–500 My, therefore these transcriptional fusion events seem to be stable and perhaps biologically advantageous. Evidence for other uORF emergence models, such as mORF fragmentation or *de novo *creation, will require further analysis of closely related organisms.

### Potential dual role for uORF proteins

uORFs can regulate specific mORF protein expression *in trans *when the *cis *uORF is intact [59,60] but it is still unclear whether uORF proteins can play additional roles in the cell. Small proteins, similar in length to uORFs, play a role in plant development and could also be involved in plant defense [61,62]. Potentially, uORFs could affect such processes independently of their role as a translational regulator. Homology group 8 uORFs are largely conserved in length, sequence, and intron position across most eukaryotes, but in fungi, algae, slime mold, and vertebrates, the associated mORF seems to be absent. The absence of the mORF and strong conservation of the uORF amino acid sequence over one billion years in these eukaryotes indicates that, in plants, this protein could act as both a regulator of mORF expression and as a *trans *acting factor in the cell.

Group 13 uORFs contain peptides similar to RS motifs found in SR proteins. SR proteins are a family of proteins required for alternative and constitutive pre-mRNA splicing [63,64]. A subset of these proteins, shuttling SR proteins, have not only been implicated in splicing but have also been shown to stimulate translation of a reporter gene when fused to the same transcript [65], analogous to a uORF-mORF associated pair. It is possible then, that group 13 uORF proteins could also play a dual role, as a translational regulator and *trans *factor.

Similarly, some uORFs in mammalian genomes might adopt these dual roles and further characterization of conserved mammalian uORFs [66] could resolve a dual role model.

### Applications

*K*_*a*_/*K*_*s *_analyses suggest that conserved peptide uORFs are under mild to strong negative selection and might therefore be useful for resolving orthology and paralogy of specific gene pairs. For example, phylogenetic studies have sometimes failed to identify all members within a uORF homology group when only considering the mORF sequence (e.g. homology group 2). Although the bHLH transcription factor domain occurs in the mORF of all three group 2 members, none were identified in the original studies, and only two of the three members have been included in the latest description of Arabidopsis bHLH family members [67-69].

Further characterization of conserved peptide uORFs and their functional mechanisms might also provide useful tools for creating inducible or repressible expression vectors in plants. AdoMetDC1, bZIP11, and PEAMT/NMT1 protein levels are regulated by conserved uORFs in a metabolite-dependent manner (polyamine, sucrose, and choline, respectively) and other conserved uORFs might also regulate mORF translation in response to cellular compounds, such as TPPases. If this is the case, further functional characterization of conserved peptide uORFs could provide the tools necessary to build constructs that are quickly inducible or repressible at the translational level under various conditions.

## Methods

### Identifying conserved uORFs in rice and Arabidopsis

Corrected RIKEN and Genoscope *Arabidopsis thaliana *ecotype Columbia and NIAS, FAIS and RIKEN *Oryza sativa *spp. *japonica *cv Nipponbare full-length cDNA collections were used for all analysis [70]. A cDNA's major ORF (mORF) was defined as the longest ORF starting with an AUG, the sequence upstream of this AUG was designated the 5' UTR, and upstream ORFs (uORFs) were any ORFs found in the 5' UTR starting with an AUG. All ORFs were identified using getorf [71]. Arabidopsis mORFs were aligned to rice cDNAs using tBLASTn with an E-value cutoff = 1e-5 [72,73] to find putative homologs. Rice cDNAs with hits below this threshold were paired with their respective Arabidopsis transcript, 5' UTR sequences extracted from both, uORFs determined using getorf, and all combinations of rice and Arabidopsis uORF peptide pairs aligned using needle [71]. The reciprocal analysis was also performed, starting with rice full-length cDNA sequences and comparing them to Arabidopsis transcript sequences. All uORFs greater than 100 amino acids were excluded from this analysis.

All pairs with scores >50 were kept and examined manually against existing Arabidopsis transcript annotations (TAIR and TIGR) and existing ESTs to determine whether aligned peptides fall within a probable 5' UTR. To validate the putative uORFs, the first 100 amino acids of the Arabidopsis mORF were aligned to Genbank plant ESTs using tBLASTn (E-value = 1e-10, limit: Viridiplantae [orgn] NOT Arabidopsis [orgn], complexity filter off), and all retrieved plant uORF sequences were aligned to rice and Arabidopsis uORFs using ClustalW [74], manually adjusted, and visualized using Jalview [75] (Figures 1, 2, 3, 4, 5, 6, 7, 8). There were two exceptions to this procedure. Because the uORFs in group 10 are 400–600 bp upstream of the mORF AUG, only the first 25 mORF amino acids were used to search Genbank plant ESTs (first 25 amino acids are very highly conserved). Secondly, high identity was limited to the 3' end of mORFs in group 17, therefore the Arabidopsis transcript's terminal 50 amino acids were aligned to Genbank non-EST plant sequences. Support for a conserved uORF was found in the *Medicago truncatula *and *Lotus corniculatus *genomic sequences.

To test whether uORFs appear upstream of non-homologous genes, Arabidopsis uORF sequences were aligned to the entire Arabidopsis genome (version 5) [76] using tBLASTn (E-value = 10). Predicted conserved uORFs were found to lie upstream of the annotated gene instead of in the annotated 5' UTR in approximately 10% of Arabidopsis and 25% of rice genes (Tables 2, 3, 4). The discrepancies with the accepted annotations, found at TAIR [76] and TIGR [77], respectively, demonstrate the benefit of using full-length cDNA sequences for this analysis.

To determine whether sequences similar to these conserved uORFs reside elsewhere in the rice and Arabidopsis genomes, uORF amino acid sequences were aligned with sequences translated from the genome sequence using tBLASTn [73]. Sequences similar to these uORFs were found within 5' UTRs of homologous mORF loci, and were absent from non-homologous transcripts, intronic regions, and intergenic regions with only one exception, Arabidopsis *NMT3 *(AGI locus identifier At1g73600). The annotated mORF for *NMT3 *[78] is not covered by any available full-length cDNA and has no EST support at its 5' end. Thus, we annotated *NMT3 *by comparison with its paralog, *NMT1 *(At3g18000) [33]. *NMT3 *possesses sequences similar to the *NMT1 *uORF, as well as sequences similar to the *NMT1 *mORF, but the TAIR annotation fuses these into a single ORF. However, *NMT3 *possesses potential splice sites that would produce transcripts with uORF and mORF sequences similar to those in NMT1. The *NMT3 *uORF predicted by one alternative splice model is the same length as, and is 72% identical to, the *NMT1 *uORF amino acid sequence (Group 13 in Figure 4).

The TAIR website was used to assign locus numbers for each Arabidopsis transcript and the TIGR website for rice locus numbers. The Arabidopsis locus numbers were then used to search for retained duplicates from the recent and ancient whole genome duplications as defined on the Arabidopsis Paralogon website [33].

### Calculating *K*_*a*_/*K*_*s*_

For homology groups 1–19, *K*_*a*_/*K*_*s *_values for homologous rice and Arabidopsis mORFs and uORFs were determined using pairwise_kaks.PLS (version 1.7) [79]. Both the approximate method (option-kaks yn00) and the maximum likelihood method (-kaks codeml) were used. Any *K*_*a*_/*K*_*s *_values resulting from a *K*_*a *_or *K*_*s *_value >10 was excluded from the analysis, as these values result in inaccurate predictions of *K*_*a*_/*K*_*s *_[80,81]. The *K*_*a*_/*K*_*s *_values for homology groups 20–26 were determined with the same approach using Arabidopsis sequences only.

### GO molecular function terms

GO molecular function terms [82] were retrieved from TAIR Locus History pages [76]. GO terms for all Arabidopsis loci were downloaded from the TAIR website and used to compare genome-wide GO molecular function term frequencies to those found in the conserved uORF-containing loci. Statistically significant differences were detected using the Exact Binomial test as described in the R program package [83]. This analysis was also carried out by GeneMerge, a program that incorporates a Bonferroni corrected *P*-value [84].

### Identification of Arabidopsis ohnologs and paralogs with conserved uORF

Conserved uORFs were found in Arabidopsis duplicates in much the same way as conserved uORFs were found between rice and Arabidopsis. uORFs and mORFs were defined in the same way, and mORF sequences were aligned to the entire Arabidopsis full-length cDNA collection using BLASTp (E-value cutoff = 1e-5) to detect transcripts deriving from a duplicated locus. mORFs aligning with ≥ 99% identity were discarded, and uORFs of all remaining pairs were aligned using needle and validated as above.

### Generation of phylogenetic trees

Sequences similar to Cox17p, Cox19p, Mrp10, CHCHD7, and uORF homology group 8 (as determined by tBLASTn and analyzed for conservation of the CHCH motif) were aligned using Muscle [85], trimmed of non-informative sites, and analyzed using Mr. Bayes v. 3.0 [86] (rates = gamma, aamodel = mixed, ngen = 2000000). Phylogenetic trees were visualized using PHYLIP's DRAWTREE program v. 3.65 [87].

Sequences similar to uORF homology group 8 were aligned, edited, and analyzed in the same manner with one exception, ngen = 3000000.

### Estimate of conserved peptide uORF prevalence

#### Number of Arabidopsis-rice loci

There is an average of 2.23 full-length cDNAs per uORF locus identified (excluding loci identified by BLAST alignment), which suggests that 15200 Arabidopsis genes are represented in the cDNA collections (34000 cDNAs/2.23 cDNAs per locus), representing approximately 60% of all Arabidopsis genes (assuming 26000 genes) [88]. In addition, Kikuchi et al [25] report that the 28000 rice full-length cDNA sequences represent 20000 transcription units (TUs) and that 64% of these (12800) have a homolog in Arabidopsis. Assuming that 60–100% of these homologs are represented in the Arabidopsis cDNA collections, the estimated number of Arabidopsis homologs screened for uORF conservation is 7800–13000. Only 80% of Arabidopsis genes also have a homolog in rice (~21000) [25], therefore the uORF-Finder program has identified 37–62% of all conserved upstream ORFs (7800/21000 to 13000/21000) when comparing rice and Arabidopsis full-length cDNAs. Therefore, there should be 61–102 loci that contain conserved uORFs: 38 loci found by uORF-Finder, 6 additional loci found by aligning known uORF sequences with the Arabidopsis genome using BLAST, and 17–58 presently unidentified loci. Using both uORF-Finder and BLAST algorithms we estimate that between 43% and 72% of conserved peptide uORFs between monocots and dicots have been identified.

#### Number of Arabidopsis-Arabidopsis loci

A total of 60% of Arabidopsis genes are represented in the full-length cDNA collections used for this study. Therefore, the probability of selecting two loci that have conserved peptide uORFs from the pool of known sequences is 0.6*0.6 = 0.36. This translates to a total of 38 loci that have conserved uORFs using an Arabidopsis-Arabidopsis comparison (14 identified (36%), and 24 unidentified).

#### Total loci

We therefore predict that there are between 99 and 140 loci in the Arabidopsis genome that contain conserved peptide uORFs, 41–58% of which have been identified.

## Abbreviations

### Species name abbreviations for Figures 1, 2, 3, 4, 5, 6, 7, 8, 9, 10, 12, and 13

Acypi, *Acyrthosiphon pisum*; Adica, *Adiantum capillus-veneris*; Ajeca, *Ajellomyces capsulatus*; Allce, *Allium cepa*; Anoga, *Anopheles gambiae*; Apime, *Apis mellifera*; Arath, *Arabidopsis thaliana*; Ascsu, *Ascaris suum*; Aspof, *Asparagus officinalis*; Betvu, *Beta vulgaris*; Bosta, *Bos taurus*; Brafl, *Branchiostoma floridae*; Brana, *Brassica napus*; Brugy, *Bruguiera gymnorhiza*; Caeel, *Caenorhabditis elegans*; Cicli, *Cicindela litorea*; Ciosa, *Ciona savignyi*; Citja, *Citrus jambhiri*; Citpa, *Citrus paradisi*; Citsi, *Citrus sinensis*; Chlre, *Chlamydomonas reinhardtii*; Cocpo, *Coccidioides posadasii*; Cryne, *Cryptococcus neoformans*; Cycru, *Cycas rumphii*; Danre, *Danio rerio*; Debha, *Debaryomyces hansenii*; Dicdi, *Dictyostelium discoidium*; Drome, *Drosophila melanogaster*; Drops, *Drosophila pseudoobscura*; Erate, *Eragrostis tef*; Escca, *Eschscholzia californica*; Eupes, *Euphorbia esula*; Eupti, *Euphorbia tirucalli*; Galga, *Gallus gallus*; Gibze, *Gibberella zeae*; Glomo, *Glossina morsitans*; Glyma, *Glycine max*; Glyso, *Glycine soja*; Gosar, *Gossypium arboreum*; Goshi, *Gossypium hirsutum*; Gosra, *Gossypium raimondii*; Haeco, *Haemonchus contortus*; Helan, *Helianthus annuus*; Hetgl, *Heterodera glycines*; Hevbr, *Hevea brasiliensis*; Homsa, *Homo sapiens *Horvu, *Hordeum vulgare*; Iponi, *Ipomoea nil*; Jugre, *Juglans regia*; Lacsa, *Lactuca sativa*; Lacse, *Lactuca serriola*; Linus, *Linum usitatissimum*; Locmi, *Locusta migratoria*; Lyces, *Lycopersicon esculentum*; Maldo, *Malus domestica*; Medtr, *Medicago truncatula*; Mescr, *Mesembryanthemum crystallinum*; Mesvi, *Mesostigma viride*; Molte, *Molgula tectiformis*; Musmu, *Mus musculus*; Neucr, *Neurospora crassa*; Nicbe, *Nicotiana benthamiana*; Oncmy, *Oncorhynchus mykiss*; Oryla, *Oryzias latipes*; Orysa, *Oryza sativa*; Parbr, *Paracoccidioides brasiliensis*; Pethy, *Petunia hybrida*; Phaac, *Phaseolus acutifolius*; Phaco, *Phaseolus coccineus*; Phypa, *Physcomitrella patens*; Pontr, *Poncirus trifoliata*; Popde, *Populus deltoides*; Popca, *Populus canadensis*; Popeu, *Populus euphratica*; Poptd, *Populus trichocarpa *× *Populus deltoides*; Poptt, *Populus tremula *× *Populus tremuloides*; Prupe, *Prunus persica*; Sacce, *Saccharomyces cerevisiae*; Sachc, *Saccharum *hybrid cultivar; Sacof, *Saccharum officinarum*; Schma, *Schistosoma mansoni*; Schpo, *Schizosaccharomyces pombe*; Selmo, *Selaginella moellendorffii*; Soltu, *Solanum tuberosum*; Sorbi, *Sorghum bicolor*; Strpu, *Strongylocentrotus purpuratus*; Strra, *Strongyloides ratti*; Styhu, *Stylosanthes humilis*; Tetni, *Tetraodon nigroviridis*; Theha, *Thelungiella halophila*; Torru, *Tortula ruralis*; Triae, *Triticum aestivum*; Trisp, *Trichinella spiralis*; Ulvli, *Ulva linza*; Ustma, *Ustilago maydis*; Vitsh, *Vitis shuttleworthii*; Vitvi, *Vitis vinifera*; Welma, *Welwitschia mirabilis*; Xenla, *Xenopus laevis*; Xentr, *Xenopus tropicalis*; Yarli, *Yarrowia lipolytica*; Zeama, *Zea mays*.

### Abbreviated species names and Genbank accession number, cDNA clone number, or genome identifier

#### Figures 1, 2, 3, 4, 5, 6, 7, 8

Group 1: Arath1 (CNS0ABWH); Arath2 (CNS09Y87); Arath3 (CNS0A364); Arath4 (CNS0A728); Arath5 (RAFL11-10-D10); Orysa1 (AK070887); Orysa2 (AK065180); Orysa3 (AK064903); Orysa4 (AK109929); Orysa5 (LOC_Os12g37410).

Group2: Arath1 (At2g31280); Arath2 (At1g06150); Arath3 (RAFL04-15-e03); Lacsa (BQ869454); Lyces (AW621910); Medtr, (BF643643); Orysa (AK074015.1).

Group3: Arath1 (CNS0A7A6); Arath2 (RAFL04-16-A04); Arath3 (RAFL09-22-L13); Cycru (CB092297); Orysa1 (AK072162); Orysa2 (AK100397); Orysa3 (AK070259); Selmo (DN838497); Torru (CN201012); Ulvli (AJ892634).

Group4: Arath1 (RAFL09-11-P17); Arath2 (RAFL09-63-H05); Arath3 (RAFL06-76-P19); Brana (CD823274); Goshi (AI730427); Gosra (CO113165); Medtr (AW689516); Orysa (AK060830); Poptt (BU896557); Prupe (BU045695).

Group 5: Arath1 (RAFL05-05-C03); Arath2 (CNS0A9PN); Gosra (CO130855); Hevbr (CB376393); Lacse (BU011020); Orysa (AK103103); Phaac (BU791117); Triae (BJ233459).

Group 6: Arath1 (RAFL05-17-I08); Arath2 (CNS0A6ZP); Aspof (CV291431); Glyma (BM143067); Gosar (BG442153); Orysa (AK064902); Pontr (CD576165); Triae (CK161649); Vitvi (CB980452).

Group 7: Arath (RAFL09-25-N17); Brana (CD836460); Mescr (BM301482); Nicbe (CK290710); Orysa1 (AK067685); Orysa2 (LOC_Os06g48350); Triae (CV066319).

Group 8: Arath (RAFL07-08-P17); Chlre (BE121764); Mesvi1 (DN255332); Mesvi2 (DN261354); Orysa (AK072620); Phypa (BJ174896); Popca (CX178804); Popeu (AJ776458); Sachc (CF573523); Triae (CA499582).

Group 9: Allce (CF443194); (Arath1 (RAFL07-09-G06); Arath2 (RAFL09-23-F23); Arath3 (At1g64140); Gosra (CO081490); Orysa1 (AK101398); Orysa2 (AK105763); Orysa3 (AK068099); Orysa4 (AK099577).

Group 10: Arath1 (RAFL07-11-O11); Arath2 (RAFL09-17-I10); Brana (CN732239); Orysa1 (AK069526); Orysa2 (AK100056); Poptt (BI131713); Sorbi (CN139168); Theha (BE758596).

Group 11: Arath1 (RAFL07-14-D12); Arath2 (CNS0A404); Glyma (CA783255); Jugre (CV197923); Medtr (AW691064); Orysa1 (AK103391); Orysa2 (AK069361); Soltu (BQ113418).

Group 12: Arath1 (RAFL07-18-F03); Arath2 (CNS0AB39); Brana (CD812479); Citse (CN185367); Jugre (CV196770); Orysa (AK060405); Popde (CK319714); Triae (BQ752938); Zeama (CD433782).

Group 13: Arath1 (RAFL08-10-M03); Arath2 (At1g48600.2); Arath3 (At1g73600); Cycru (CB093136); Gosra (CO080661); Iponi (BJ562806); Linus (CA483285); Medtr (AW587372); Orysa1 (LOC_Os05g47540); Orysa2 (AK102037); Phypa (BJ204269); Xenla (CA792398); Xentr (CX412233); Zeama (AY103779).

Group14: Allce (CF450799); Arath (RAFL09-10-M04); Medtr (AW267817); Nicbe (CK295530); Orysa (AK101569); Soltu (CK258175); Zeama (CO519993). Group 15: Adica (BP914226); Arath1 (CNS0ADY7); Arath2 (RAFL08-17-G21); Arath3 (RAFL04-17-N21); Arath4 (RAFL16-69-M04); Citpa (DN959636); Gosra (CO125506); Maldo (CV082382); Medtr (CX528608); Orysa1 (AK102703); Orysa2 (AK101749); Orysa3 (AK071582); Orysa4 (AK065674); Sacof (CA154823); Vitvi (CB001711); Welma (DT579937).

Group 15: Adica (BP914226); Arath1 (CNS0ADY7); Arath2 (RAFL08-17-G21); Arath3 (RAFL04-17-N21); Arath4 (RAFL16-69-M04); Citpa (DN959636); Gosra (CO125506); Maldo (CV082382); Medtr (CX528608); Orysa1 (AK102703); Orysa2 (AK101749); Orysa3 (AK071582); Orysa4 (AK065674); Sacof (CA154823); Vitvi (CB001711); Welma (DT579937).

Group 16: Arath (CNS0A4RC); Medtr (AW693231); Orysa1 (AK071885); Orysa2 (AK067447).

Group 17: Arath1 (RAFL09-25-E19); Arath2 (At5g03190); Arath3 (RAFL19-67-G09); Arath4 (At5g01710); Gosra (CO108440); Lyces (AW738430); Medtr1 (BQ149694); Medtr2 (AC144517); Orysa1 (AK69088); Orysa2 (AK070250); Sacof (CA191644).

Group 18: Arath (RAFL08-18-B11); Gosra (CO115325); Nicbe (CK286574); Orysa (AK061433).

Group 19: Arath (CNS09ZXM); Eupes (DV113097); Helan (AJ541596); Medtr (BI309364); Orysa (AK068270); Triae (CD927685); Vitvi (CB918939); Zeama (DV166198).

Group 20: Arath1 (RAFL04-17-G13); Arath2 (CNS0A8YX); Brana (CD835762); Brugy (BP941533); Gosar (BF274209); Maldo (CN940921); Medtr (BE316669); Styhu (L36823).

Group 21: Allce (CF450138); Arath1 (RAFL07-08-G04); Arath2 (RAFL21-49-G19); Betvu (BQ594525); Brana (CD835573); Erate (DN481483); Escca (CD481239); Eupti (BP958766); Gosra (CO074819); Glyma (BU761432); Horvu (AV834976); Lacse (BQ998418); Maldo (CV881926); Medtr (CA991201); Orysa (AK100575); Popca (CX182168).

Group 22: Arath1 (RAFL07-11-D20); Arath2 (RAFL11-03-J07); Brana (CD836422); Horvu (CA023398); Orysa (CK041713); Sacof (CA242575); Triae (BJ247925); Zeama (CO458204).

Group 23: Arath1 (RAFL07-11-L03); Arath2 (RAFL09-07-L11); Citsi (CV720092); Glyma (BI892512).

Group 24: Arath1 (RAFL07-14-J09); Arath2 (CNS0A44P); Brana (CD828343); Glyma (BI471587); Horvu (BQ471053); Orysa (AK119634); Sacof (CA118382); Sorbi (CB928687); Triae (CA483985); Zeama (CO520078).

Group 25: Arath1 (RAFL09-94-P19); Arath2 (CNS0A6N0); Brana (CD835519); Citsi (CN191447); Escca (CD481312); Glyma (BE805986); Phaco (CA913939); Soltu (DN940765); Vitsh (CV098492).

Group 26: Arath1 (CNS0A7NI); Arath2 (CNS0A1F5); Citja (CO912573); Pethy (CV298852); Poptd (CN521002); Prupe (BU045483).

#### Figure 9

Arath (RAFL07-08-P17); Caeel (U10402); Ciosa (BW577210); Danre (CO350578); Dicdi (AU072562); Drome (AI297387); Homsa (BU541024); Mesvi (DN255332); Neucr (BX284746); Orysa, (AK072620); Phypa (BJ174896); Strpu (CX079489); Ustma (CF644197).

#### Figure 10

Arath1 (RAFL08-10-M03); Arath2 (At1g48600.2); Arath3 (At1g73600); Cycru (CB093136); Gosra (CO080661); Iponi (BJ562806); Linus (CA483285); Medtr (AW587372); Orysa1 (LOC_Os05g47540); Orysa2 (AK102037); Phypa (BJ204269); Xenla (CA792398); Xentr (CX412233); Zeama (AY103779).

#### Figure 12

Acypi (CV847404); Ajeca (CV605785); Anoga1 (BX617953), Anoga2 (XM_552406); Apime (NW_622706); Arath1 (BP562704), Arath2 (AY065264), Arath3 (RAFL07-08-P17), Arath4 (NM_179521), Arath5 (NM_112400); Ascsu (BM964977); Bosta (CO877216); Brafl1 (BW786058), Brafl2 (BW840607); Caeel (U10402); Chlre1 (BE121764), Chlre2 (AF280543); Cicli (CV156944); Ciosa (BW577210); Cocpo (CO006101); Cryne (XM_572394); Danre (CO350578); Debha (NC_006045); Dicdi1 (AU072562), Dicdi2 (XM_631387); Drome1 (AI297387), Drome2 (AY102691); Drops (DR121964), Erate (DN481021); Galga1 (BX935835), Galga2 (CR407540); Gibze (BI750032); Glomo (BX557417); Glyso (BG045953); Haeco (CA956938); Hetgl (CB299856); Homsa1 (DR155443), Homsa2 (CR607136), Homsa3 (BU541024), Homsa4 (AY957566), Homsa5 (NM_005694); Hordvu (BF628344); Locmi1 (CO854527), Locmi2 (CO825844); Mesvi1 (DN255332), Mesvi2 (DN261354); Molte (CJ368011); Musmu1 (BC030366), Musmu2 (AK010111); Neucr (BX284746); Oncmy (BX081024); Oryla (BJ737531); Orysa1 (XM_482456), Orysa2 (AK072620), Orysa3 (AK120143), Orysa4 (XM_468245); Parbr (CA581923); Phypa1 (BJ966696), Phypa2 (BJ174896); Popca (CX178804); Popeu (AJ776458); Sacce1 (NC_001136), Sacce2 (AY692601), Sacce3 (NC_001144), Sacce4 (NC_001144); Sachc (CF573523); Schma (CD081475); Schpo1 (NM_001019463), Schpo2 (NM_001022867), Schpo3 (NM_001022571); Sorbi (CD423660); Strpu (CX079489); Strra (BI323578); Tetni1 (CR709012), Tetni2 (CNS0G27U); Triae (CA499582); Trisp (BQ693345); Ustma1 (CF644197), Ustma2 (XM_754796); Xenla1 (BI477811), Xenla2 (BC084847); Xentr1 (BC075310), Xentr2 (CN119217); Yarli (XM_500713).

#### Figure 13

Acypi (CV847404); Ajeca (CV605785); Anoga (BX617953); Apime (NW_622706); Arath (RAFL07-08-P17); Ascsu (BM964977); Bosta (CO877216); Brafl1 (BW840607), Brafl2 (BW786058); Caeel (U10402); Chlre (BE121764); Cicli (CV156944); Ciosa (BW577210); Danre (CO350578); Debha (NC_006045); Dicdi (AU072562); Drome (AI297387); Drops (DR121964); Galga (CR407540); Gibze (BI750032); Glomo (BX557417); Haeco (CA956938); Hetgl (CB299856); Homsa (BU541024); Locmi (CO825844); Mesvi1 (DN255332), Mesvi2 (DN261354); Molte (CJ368011); Musmu (AK010111); Neucr (BX284746); Oncmy (BX081024); Oryla (BJ737531); Orysa (AK072620); Phypa (BJ174896); Popca (CX178804); Popeu (AJ776458); Sachc (CF573523); Schma (CD081475); Sorbi (CD423660); Strpu (CX079489); Strra (BI323578); Tetni (CR709012); Triae (CA499582); Trisp1 (BQ693345), Trisp2 (BQ692350); Ustma (CF644197); Xenla (BI477811); Xentr (CN119217).

## Authors' contributions

Both CAH and RAJ designed and implemented the analyses for the present study. CAH drafted the manuscript and RAJ provided critical comments. Both authors have read and approved the final manuscript.

## Supplementary Material

Additional file 1 Alignment used to generate Figure 12 Click here for file

Additional file 2 Alignment used to generate Figure 13 Click here for file
