# Supplementary material for: Identification of novel conserved peptide uORF homology groups in Arabidopsis and rice reveals ancient eukaryotic origin of select groups and preferential association with transcription factor-encoding genes
Source: BMC Biol. 2007 Jul 30;5:32. doi: 10.1186/1741-7007-5-32 (PMC2075485; doi:10.1186/1741-7007-5-32)
Supplement: Additional file 1 — Alignment used to generate Figure 12 [file 1741-7007-5-32-S1.doc]

Additional File 1. Alignment used to generate phylogeny in Figure 12.

Hordvu MGRKA-GLYINPKKFGGVVK PCMLEMTDFLNCLAL--NKH IDEKCTRHKELLITCAKTIN YHL

Orysa1 MGRKAGALYINPKKFGGGAK PCMIEMVSFLNCLAL--NKQ NDDKCVRQKDLLVACTKTIN YHL

Sacce1 PPVYRLPPLPRLKVKKQEAN KCLVLMSNLLQCWSS--YGH MSPKCAGLVTELKSCVQKSN INY

Arath1 AQPSK--------------E PCKKEACDIQACLSK--NNF LPQRCQRVIEMLQACCERCN NES

Schpo1 VNPPRLQGLSRLRVRPKNTI PCGQEMAALLGCWQNHGGQT DTAQCANLVAALENCMKTTH RKT

Cocpo MKPVRLQTIQSLRIQRQEQS PCQTAMSAVLNCWAS--AGH NIQGCHALEDQLRQCMDTAK YQK

Phypa -MGET-DAPARTGPLKLSDS MCAPQYAASLKCLDE--ANY DKSKCQDHFDAYKECLRLEA NRK

Arath2 GAAAAYPSAARI-----SDS PCYLQYSASLKCLEE--FGS DKSKCQDHFDVYKECKKKER EAR

Erate DSPTHRAYSPATSNPPIADS ACFPQYTASLKCLE---AHQ DKSKCQQQFDDYKECKKKER EAR

Yarli GPDDPTAMSNRVQFLAQYYD PCAEASKMSLNCLER--NNY KKAMCEEYFQIYRDCGEIFE TEN

Parbr WEKAK----PMFTNKPGYYD PCQDFADRSIKCMRR--NGN DKTMCSDYFQAYRDCWTTQR KNK

Sacce2 ENPVN-KYKFALKADSQYYD PCEESSKLSFQCLER--NDY DRSKCQEYFDAYRECLTARR KNR

Locmi1 SKQSRASQRKKDQLNNADNN PCLKEHELSLKCLSD--SNY DRDACSKHFDNYNICRAKGI IPY

Homsa2 MPSVT----QRLRDPD--IN PCLSESDASTRCLDE--NNY DRERCSTYFLRYKNCRKNGV KPF

Musmu1 PMVTR-----RLRDPD--IN PCLSESDASTRCMDE--NNY DRERCSSYFLKYKNCRQNGV QPS

Dicdi1 -WSQRV----------ASLG DCTFEMSIYGACVTSNLDNI EKNVCKVEFEKFKNCMAKSM VSK

Chlre1 PPGLRSAPEQTAARIGRAFA ACSAKAELYGTCIKKLVPEV DKGVCAKEFQELKTCFTRAM RSG

Mesvi1 GGRSVPSSSKYLKELGQGLA SCTAEVAVYGKCISQGLQDI NKGMCEQEFRALTKCMRQAR VGR

Mesvi2 -----PSSWKILKELRQVWG LCPPEVAVYRKCISQALQDI NKGMCEQDFRALTKCMRQAR VGR

Acypi MDAVN-KAKSRFKRYPELLL TCRVEGLEYAACIIKNEKDL KPNSCKTQFMKFRECLTSNA LKL

Schma MPVVL-DGKTRLLRYPALLS QCILESSVYAKCVLSL-KDV KHNYCEKEFQILKRCVQSRA RET

Locmi2 MEAVQ-AARSRLRKYPLLLS KCSAEASLYATCVLSR-DNI KHSECEHEFRKLTTCLQKAA QEA

Cicli MESVK-KTQNRLRQYPVLLG KCSSEAIAYANCVLQK-DSV NHNDCLQDFKKFKLCLQKTA SEL

Apime MEAVK-KAKERFRKYPIIVA QCHESGAKYAACVLAK-SNL RKDDCENEFKEFKACLMKAA AKN

Drops MESVR-KANQRLRNYPILLT KCADKASAYAICVSRD-LNV QHKICDAEFKEFLSCIRKSA MEL

Drome1 MESVR-KANQRIRNYPVLLS KCADKATAYAVCVSRD-LNV QHKICDTEFKEFLSCIRKTA LEM

Glomo MESVR-KANRRLRNYPILLS KCAHSAAIYAACVTRD-LNI EYRTCEKEFKLFKECLQKAA KDM

Anoga1 MESVK-RANQRLRSYPLLMA KCSVAAAAYATCVTTD-LNV AHRSCDKEFNNFKECMRKAA IEM

Ciosa QSVLR-AQNYLRRELPVKVK SCSKQAVAYGTCVGEW-DNL RKGDCEKEFLAFKQCIQSLK K--

Molte YPYLR-GQRYIFKELPASIK LCSKESFLYGKCVFEW-DNL RKNDCLKEFNSLKHCVRNIK K--

Brafl1 MKPTR-PLARPIHKMSEALV GCGREAIVYGNCVNSW-QDI QKGDCRREFEHFKNCYRKAL S--

Ustma1 PGAIKQRDVAPVQTFAKAAA KCASEARIYGACVTANYENI ERNMCQKEFFAFKACVQQKL GRK

Phypa2 SEKGR-PLPSPLK---NVFL RCSPAMKEYGQCVATKLPAV EKGMCEKEFLALKTCMQNAA KKK

Orysa2 -MKER-NPAVASSALARILA ACASQAKDYGRCIAEKVPEI EQNMCAKEFLALRSCMQTVV KRK

Sachc -MKER-KPAASSPALVRILA VCASQAKDYGRCIAAKIPEI EHNMCSKEFLALRACMQTAV KNK

Sorbi -MKER-KPAASSPALVRILA VCASQAKDYGRCIAAKVPEI EHHMCSKEFLALRACMQTVV KNK

Triae -MKER-KPAAPS-ALARILA TCASQAKDYGRCIAAKVPEI EHNMCSKEFLALRACMQTAV KNK

Arath3 ---MKEKNSTTASTLGRILA TCSKQAKDYGSCVASKVHEV ERDICLKEFLALKSCMQHTI RGK

Popca -MKER-NTTSTLR---RVLV NCAAQAKEYGGCVAAKVPEI ERDMCLKEFLALKNCMQNTI RGK

Popeu -MKERNSSSSTLR---RILV NCAAQAKEYGCCVAEKVPEI ERDMCLKEFLALKNCMHITI RGK

Strpu SGSVQ-KARQKMAQFPAAFA ECTPQALAYGRCVSSK-DHV GRNDCGKEFQTYKECLQKAM SKI

Ajeca TGRTR-----PIEKFAKATA QCSAQASAYGKCVFADYNAI RKDMCAKEFMKLKECYLV-- ---

Gibze QQRTR-----PIQKLAKAVS QCSVEATSYGKCIVADYNAV HKDKCVKEFMRLKDCYLAAS KKS

Neucr MQPPK-TTVRPIQRFASAVS KCSVESAAYGKCILADYNSV HKDMCVKEFMRLKDCYLVRS SPP

Xentr2 SGKITAPGKGALGRIPQLLA KCRVQALSYGKCVSAAAEEL RRGACAKEFEDLKQCMIMAA KGK

Xenla1 ARGATGPGKGALDRIPQLLA KCRVQALAYGKCASAAADEL RRGTCAKEFADLKECMIMAT KRK

Oncmy WTRSR----ERMRRFPELFA QCSGEAAAYGKCVTATTQEL RKDLCVKEFDALKTCIVTAA KKG

Danre WTRSR----ARMRLFPELMA QCSGEATAYGKCVAATTQEL TRNMCVKEFEALKSCFQSAA KKA

Tetni WTRSR----DKLKMFSELFA ECSLEAAAYGKCVAATTREL KKDVCSKEFGALKTCFMDAA KKK

Oryla WTRSR----EKMRNFSDIFS KCADEAAAYGKCVAATTQEL KKDLCAKEFEALKTCFVKAA KRH

Galga2 WLRAR----ARLRRFPALLA GCGEQASAYGRCVAAASAEL RRDVCLREFQALRECFARAA AAT

Musmu2 WGRVR----SRFRAFPEHLA ACGAEASAYGKCVQASTGRL SKDLCVREFEALRSCFAAAA KKT

Bosta WGRVR----SRLRAFPECLA ACGAEAAAYGRCVQASTGCL KKDLCAQEFEALRSCFAAAA KNT

HOmsa3 WGRVR----SRLRAFPERLA ACGAEAAAYGRCVQASTGRL SKDFCAREFEALRSCFAAAA KKT

Brafl2 MRDLS-HVRRRIRQFSYAMS ECSPQVTAYGRCVAVK-ENI KKGDCAKEFQAMKDCARKVV QKS

Strra -MTDR----QKLLKYSKYLA NCTSEISLYGTCVSTKSDKI SKGDCTKEFEALFRCVEKQI RTA

Caeel MPKTQ----QRLLNYATSIA KCPTETSNYGSCVSVQAERI KQGDCSAEFRKLIDCVTKNL KKK

Haeco -------------QFASTFA ACPPEAAAYGACVSRQAERI TKDACANEFSKLLDCVKKQK NKA

Ascsu MPNEQ-----RLLKYAIYLS NCSPEALNYGRCVAEKAEKV TKSACEKEFSLLLKCFKKEV KAA

Hetgl -MREK----KRLLKFAEYLG HCSREATDYGKCVATNAERI RKGDCSTEFDRLIACLKKRQ VRL

Trisp -MQKR-----RILSFAEHLS ACSHQCAAYGRCVSKHAEDI RKGVCEKEFLELIACIRKKP KLS

Ustma2 FSDFK-VSPPERGSFPDHDG ECKSVMQEYMNCIKY--NRN DNGKCRHLSRAYLQCMDNLG FKD

Sacce3 LSALR-PTPPERGSFPDHDG ECTKYMQEYLKCMQLV-QNE NAMNCRLLAKDYLRCWSHLG LPE

Drome2 QKKFV-PTPPEKGSFPDHEG LCKKQFLLYASCLRK--NAQ DTSQCRQDAQNYLACWSKLG FHD

Tetni2 SKSFQ-PRPPDKGSFPDHFG ECTAFKERFMACLRE--KGF DNSKCRMQSKEYLECLEKLG FKD

Homsa4 TKSFQ-PRPPDKGSFPDHLG ECKSFKEKFMKCLHN--NNF ENALCRKESKEYLECLEKLG FGD

Schopo2 -MTTR--EPPERGSFPDHFG ECTHVMKQYLECIKV--KRE NQEECRLLAKKYLQCMKNLG FHG

Arath4 NRGLR-PIPPEKGIFPDHLH ECDAEKKEYLGCLKS--SAH KSEQCRHLSKKYLQCMAELG FSG

Glyso NRGLR-PVPPEKGIFPDHMH LCDLEKIEYLNCLKT--AGH QSEKCRLFSKKYLQCLELGF KES

Orysa3 NRGVR-PVPPEKGVFPDHLH ECDLEKKDYLACLKS--TGF QSEKCRQFSKKYLECMSELG FRS

Sacce4 DKKQEQENHAECEDKPKPCC VCKPEKEERDTCILF--NGQ DSEKCKEFIEKYKECMKGYG FEV

Schopo3 ATKVSEPAPIASEEKPKPCC ACPETKQARDACMLQ--SSN GPIECAKLIEAHKKCMAQYG YEV

Cryne PTSSS-PDATAKEVNPKPCC ACPETKQARDDCFIKSAPGE GETNCRDFIEAHKACMRGYG FKV

Anoga2 VDTSSTPATTKEKPKCKACC ACPETKRARDACI----MEN GEEKCSELIEKHKQCMRDMG FNI

Homsa5 DSNPA-PPESQEKKPLKPCC ACPETKKARDACI----IEK GEEHCGHLIEAHKECMRALG FKI

Xenla2 CESLS-PSAESQEKKPKPCC ACPETKKARDACI----IEN GEEKCQHLIEAHKECMRSLG FKV

Chlre2 LPTPS-PAPPGVPIGPKICC SCPDTKKLRDTCIAE--RGE EHAYCQALIEAHKACLRVEG FKV

Arath5 SEPSKAAASAETKPKKRICC ACPDTKKLRDECI----VEH GESACTKWIEAHKICLRAEG FNV

Orysa4 SEGGSAAPAPATDSKPKICC ACPDTKRLRDECI----VEH GESACTKWIEAHKRCLRAEG FNV
