# Supplementary material for: Identification of novel conserved peptide uORF homology groups in Arabidopsis and rice reveals ancient eukaryotic origin of select groups and preferential association with transcription factor-encoding genes
Source: BMC Biol. 2007 Jul 30;5:32. doi: 10.1186/1741-7007-5-32 (PMC2075485; doi:10.1186/1741-7007-5-32)
Supplement: Additional file 2 — Alignment used to generate Figure 13 [file 1741-7007-5-32-S2.doc]

Additional File 2. Alignment used to generate phylogeny in Figure 13.

Chlre -AARIGRAFAACSAKAELYG TCIKKLV---PEVDKGVCAK EFQELKTCFTRAMRSGSGRA

Mesvi1 YLKELGQGLASCTAEVAVYG KCISQGL---QDINKGMCEQ EFRALTKCMRQARVGR----

Mesvi2 ILKELRQVWGLCPPEVAVYR KCISQAL---QDINKGMCEQ DFRALTKCMRQARVGR----

Dicdi KWSQRVASLGDCTFEMSIYG ACVTSNL---DNIEKNVCKV EFEKFKNCMAKSMVSKVKK-

Schma RLLRYPALLSQCILESSVYA KCVLSL----KDVKHNYCEK EFQILKRCVQSRARETGIRL

Locmi RLRKYPLLLSKCSAEASLYA TCVLSR----DNIKHSECEH EFRKLTTCLQKAAQEA----

Cicli RLRQYPVLLGKCSSEAIAYA NCVLQK----DSVNHNDCLQ DFKKFKLCLQKTASELKIKI

Apime RFRKYPIIVAQCHESGAKYA ACVLAK----SNLRKDDCEN EFKEFKACLMKAAAKNNIRL

Drops RLRNYPILLTKCADKASAYA ICVSRDL----NVQHKICDA EFKEFLSCIRKSAMELKTKL

Drome RIRNYPVLLSKCADKATAYA VCVSRDL----NVQHKICDT EFKEFLSCIRKTALEMKTKL

Glomo RLRNYPILLSKCAHSAAIYA ACVTRDL----NIEYRTCEK EFKLFKECLQKAAKDMKTKL

Anoga RLRSYPLLMAKCSVAAAAYA TCVTTDL----NVAHRSCDK EFNNFKECMRKAAIEMKTKL

Brafl2 PIHKMSEALVGCGREAIVYG NCVNSW----QDIQKGDCRR EFEHFKNCYRKALS------

Ciosa LRRELPVKVKSCSKQAVAYG TCVGEW----DNLRKGDCEK EFLAFKQCIQSLKK------

Molte IFKELPASIKLCSKESFLYG KCVFEW----DNLRKNDCLK EFNSLKHCVRNIKK------

Ustma PVQTFAKAAAKCASEARIYG ACVTANY---ENIERNMCQK EFFAFKACVQQKLGRKW---

Phypa LPSPLKNVFLRCSPAMKEYG QCVATKL---PAVEKGMCEK EFLALKTCMQNAAKKK----

Orysa ASSALARILAACASQAKDYG RCIAEKV---PEIEQNMCAK EFLALRSCMQTVVKRKA---

Sachc SSPALVRILAVCASQAKDYG RCIAAKI---PEIEHNMCSK EFLALRACMQTAVKNKV---

Sorbi SSPALVRILAVCASQAKDYG RCIAAKV---PEIEHHMCSK EFLALRACMQTVVKNKA---

Triae APSALARILATCASQAKDYG RCIAAKV---PEIEHNMCSK EFLALRACMQTAVKNKA---

Arath TASTLGRILATCSKQAKDYG SCVASKV---HEVERDICLK EFLALKSCMQHTIRGKA---

Popca TTSTLRRVLVNCAAQAKEYG GCVAAKV---PEIERDMCLK EFLALKNCMQNTIRGKA---

Popeu SSSTLRRILVNCAAQAKEYG CCVAEKV---PEIERDMCLK EFLALKNCMHITIRGKA---

Strpu KMAQFPAAFAECTPQALAYG RCVSSK----DHVGRNDCGK EFQTYKECLQKAMSKIKK--

Ajeca PIEKFAKATAQCSAQASAYG KCVFADY---NAIRKDMCAK EFMKLKECYLV---------

Gibze PIQKLAKAVSQCSVEATSYG KCIVADY---NAVHKDKCVK EFMRLKDCYLAASKKS----

Neucr PIQRFASAVSKCSVESAAYG KCILADY---NSVHKDMCVK EFMRLKDCYLVRSSPPSLFT

Xentr ALGRIPQLLAKCRVQALSYG KCVSAAASGREELRRGACAK EFEDLKQCMIMAAKGKNK--

Xenla ALDRIPQLLAKCRVQALAYG KCASAAAAGRDELRRGTCAK EFADLKECMIMATKRKMK--

Oncmy RMRRFPELFAQCSGEAAAYG KCVTATTTGRQELRKDLCVK EFDALKTCIVTAAKKGVK--

Danre RMRLFPELMAQCSGEATAYG KCVAATTTGKQELTRNMCVK EFEALKSCFQSAAKKAVK--

Tetni KLKMFSELFAECSLEAAAYG KCVAATTTGTRELKKDVCSK EFGALKTCFMDAAKKKGK--

Oryla KMRNFSDIFSKCADEAAAYG KCVAATTAGRQELKKDLCAK EFEALKTCFVKAAKRHGR--

Galga RLRRFPALLAGCGEQASAYG RCVAAASAGSAELRRDVCLR EFQALRECFARAAAATKC--

Musmu RFRAFPEHLAACGAEASAYG KCVQASTAPGGRLSKDLCVR EFEALRSCFAAAAKKTMMGG

Bosta RLRAFPECLAACGAEAAAYG RCVQASTAPGGCLKKDLCAQ EFEALRSCFAAAAKNTLTGG

Homsa RLRAFPERLAACGAEAAAYG RCVQASTAPGGRLSKDFCAR EFEALRSCFAAAAKKTLEGG

Brafl1 RIRQFSYAMSECSPQVTAYG RCVAVK----ENIKKGDCAK EFQAMKDCARKVVQKSH---

Strra KLLKYSKYLANCTSEISLYG TCVSTKS---DKISKGDCTK EFEALFRCVEKQIRTAKK--

Caeel RLLNYATSIAKCPTETSNYG SCVSVQA---ERIKQGDCSA EFRKLIDCVTKNLKKK----

Haeco ---QFASTFAACPPEAAAYG ACVSRQA---ERITKDACAN EFSKLLDCVKKQKNKAK---

Ascsu RLLKYAIYLSNCSPEALNYG RCVAEKA---EKVTKSACEK EFSLLLKCFKKEVKAANK--

Hetgl RLLKFAEYLGHCSREATDYG KCVATNA---ERIRKGDCST EFDRLIACLKKRQVRL----

Trisp1 RILSFAEHLSACSHQCAAYG RCVSKHA---EDIRKGVCEK EFLELIACIRKKPKLSRSHR

Trisp2 RILSFAEHLSACSYQCAAYG RCVSKHA---EDIRKGVCEK EFLELIACIRKKSKLSRSHR
